# The *Escherichia coli* Phosphotyrosine Proteome Relates to Core Pathways and Virulence

**DOI:** 10.1371/journal.ppat.1003403

**Published:** 2013-06-13

**Authors:** Anne-Marie Hansen, Raghothama Chaerkady, Jyoti Sharma, J. Javier Díaz-Mejía, Nidhi Tyagi, Santosh Renuse, Harrys K. C. Jacob, Sneha M. Pinto, Nandini A. Sahasrabuddhe, Min-Sik Kim, Bernard Delanghe, Narayanaswamy Srinivasan, Andrew Emili, James B. Kaper, Akhilesh Pandey

**Affiliations:** 1 Department of Microbiology and Immunology, University of Maryland School of Medicine, Baltimore, Maryland, United States of America; 2 Institute of Bioinformatics, International Tech Park, Bangalore, India; 3 McKusick-Nathans Institute of Genetic Medicine and Department of Biological Chemistry, Johns Hopkins University, Baltimore, Maryland, United States of America; 4 Manipal University, Manipal, India; 5 Banting and Best Department of Medical Research, Terrence Donnelly Center for Cellular and Biomolecular Research, University of Toronto, Toronto, Canada; 6 Department of Biology, Wilfrid Laurier University, Waterloo, Canada; 7 Molecular Biophysics Unit, Indian Institute of Science, Bangalore, India; 8 Thermo Fisher Scientific (Bremen) GmbH, Bremen, Germany; 9 Department of Pathology and Oncology, Johns Hopkins University, Baltimore, Maryland, United States of America; Collège de France, France

## Abstract

While phosphotyrosine modification is an established regulatory mechanism in eukaryotes, it is less well characterized in bacteria due to low prevalence. To gain insight into the extent and biological importance of tyrosine phosphorylation in *Escherichia coli*, we used immunoaffinity-based phosphotyrosine peptide enrichment combined with high resolution mass spectrometry analysis to comprehensively identify tyrosine phosphorylated proteins and accurately map phosphotyrosine sites. We identified a total of 512 unique phosphotyrosine sites on 342 proteins in *E. coli* K12 and the human pathogen enterohemorrhagic *E. coli* (EHEC) O157:H7, representing the largest phosphotyrosine proteome reported to date in bacteria. This large number of tyrosine phosphorylation sites allowed us to define five phosphotyrosine site motifs. Tyrosine phosphorylated proteins belong to various functional classes such as metabolism, gene expression and virulence. We demonstrate for the first time that proteins of a type III secretion system (T3SS), required for the attaching and effacing (A/E) lesion phenotype characteristic for intestinal colonization by certain EHEC strains, are tyrosine phosphorylated by bacterial kinases. Yet, A/E lesion and metabolic phenotypes were unaffected by the mutation of the two currently known tyrosine kinases, Etk and Wzc. Substantial residual tyrosine phosphorylation present in an *etk wzc* double mutant strongly indicated the presence of hitherto unknown tyrosine kinases in *E. coli*. We assess the functional importance of tyrosine phosphorylation and demonstrate that the phosphorylated tyrosine residue of the regulator SspA positively affects expression and secretion of T3SS proteins and formation of A/E lesions. Altogether, our study reveals that tyrosine phosphorylation in bacteria is more prevalent than previously recognized, and suggests the involvement of phosphotyrosine-mediated signaling in a broad range of cellular functions and virulence.

## Author Summary

While phosphotyrosine modification is established in eukaryote cell signaling, it is less characterized in bacteria. Despite that deletion of bacterial tyrosine kinases is known to affect various cellular functions and virulence of bacterial pathogens, few phosphotyrosine proteins are currently known. To gain insight into the extent and biological function of tyrosine phosphorylation in *E. coli*, we carried out an in-depth phosphotyrosine protein profiling using a mass spectrometry-based proteomics approach. Our study on *E. coli* K12 and the human pathogen enterohemorrhagic *E. coli* (EHEC) O157:H7, which is a common cause of food-borne outbreaks of diarrhea, hemorrhagic colitis and hemolytic uremic syndrome, reveal that tyrosine phosphorylation is far more prevalent than previously recognized. Target proteins are involved in a broad range of cellular functions and virulence. Proteins of the type III secretion system (T3SS), required for the attaching and effacing lesion phenotype characteristic for intestinal colonization by EHEC, are tyrosine phosphorylated. The expression of these T3SS proteins and A/E lesion formation is affected by a tyrosine phosphorylated residue on the regulator SspA. Also, our data indicates the presence of hitherto unknown *E. coli* tyrosine kinases. Overall, tyrosine phosphorylation seems to be involved in controlling cellular core processes and virulence of bacteria.

## Introduction

Protein phosphorylation is an evolutionarily highly conserved post-translational modification important for signal transduction in living organisms. The ability of bacteria to rapidly adapt to changing environments, crucial for survival and successful infection of the host by bacterial pathogens, relies on an extensive regulatory network also involving protein phosphorylation. Reversible protein phosphorylation targeting arginine, aspartate, histidine, serine, threonine and tyrosine residues is highly integrated in regulatory networks of bacteria. Among these, phosphorylation-mediated signaling through histidine and aspartate in bacterial two-component systems is the best characterized [1]. Phosphorylation on serine/threonine/tyrosine (Ser/Thr/Tyr) residues was initially associated with signaling in eukaryotes; however, during the past two decades it has emerged as an important regulatory function in prokaryotes as well. Recent high resolution mass spectrometry-based phosphoproteomic studies have unambiguously identified phosphorylation events in bacteria on Ser, Thr and less frequently on Tyr residues [2], significantly expanding the repertoire especially of Ser and Thr phosphorylated proteins. Notably, a comprehensive phosphoproteomic analysis of *Mycobacterium tuberculosis* revealed more than 500 phosphorylation events on Thr/Ser but none on Tyr residues [3]. About 121 phosphotyrosine (pTyr) sites have so far been reported on 114 proteins in 11 bacterial species by phosphoproteomics studies [4]. While the role of pTyr modification in eukaryotes is well established in cell growth, proliferation and differentiation [5], its role is less clear in bacteria.

The Gram-negative bacterium *Escherichia coli* comprises diverse isolates ranging from gastrointestinal commensals to various disease-causing ones including the human pathogen enterohemorrhagic *E. coli* (EHEC) [6]. EHEC serotype O157:H7, a commonly occurring food-borne pathogen in developed countries, is associated with diarrhea, hemorrhagic colitis and the potentially fatal hemolytic uremic syndrome [7]. Virulence of EHEC O157:H7 is in part attributed to the presence of about 53 pathogenicity islands (PAI) containing genes that are absent in non-pathogenic *E. coli* K12 [8], [9]. EHEC O157:H7 infection is characterized by an attaching and effacing (A/E) histopathological lesion phenotype of infected intestinal epithelial cells, which is due to the activity of a type III secretion system (T3SS), mainly encoded by the locus of enterocyte effacement (LEE) PAI [10]. The T3SS apparatus, responsible for the translocation of bacterial effector proteins into host cells, consists of a needle-like structure composed of the EspA filament, and additional translocon components such as EspB and EspD that form a pore in the host cell membrane. Among the proteins translocated by the T3SS are translocated intimin receptor Tir which binds to the outer membrane adhesion intimin [6], [11]. It is well-established that host cell tyrosine kinases phosphorylate T3SS effector proteins including Tir from enteropathogenic *E. coli* (EPEC) and the mouse pathogen *Citrobacter rodentium* upon infection to subsequently manipulate host signaling pathways and induce actin rearrangements [12], [13]. However, phosphotyrosine modification of virulence-associated *E. coli* proteins by bacterial tyrosine (BY) kinases has yet to be demonstrated.

There are currently two characterized classical BY kinases in *E. coli*, Etk and Wzc, which are structurally and functionally different from eukaryotic tyrosine kinases [14]. BY kinases are associated with exopolysaccharide synthesis, antibiotic resistance, phage lysogenization and the heat shock response [15]. They affect virulence mainly through their involvement in capsular synthesis [16], [17]. Although *E. coli* is among the best characterized bacterial species, the extent of known phosphotyrosine modifications by bacterial kinases is currently limited to about 32 proteins. A global phosphoproteome analysis of *E. coli* K12 using a metal oxide affinity-based phosphopeptide enrichment approach revealed 74 unique Ser/Thr phosphorylation sites, whereas only 7 Tyr phosphorylation sites were identified [18], implying that specifically enriching for phosphotyrosine-modified phosphopeptides could expand the coverage of the phosphotyrosine-modified proteome in *E. coli*. Our current knowledge on tyrosine phosphorylation suggests biological significance, yet, a comprehensive identification of phosphotyrosine proteins is required to better understand the function of tyrosine phosphorylation in bacteria.

To gain further insight into the extent and biological role of tyrosine phosphorylation in *E. coli*, we took advantage of technological progress in mass spectrometry-based proteomics and immunoaffinity enrichment strategies, recently applied in eukaryotic phosphotyrosine proteome studies, which enable comprehensive identification of phosphotyrosine events and accurate mapping of phosphorylation sites [19], [20]. Here, we present an in-depth phosphotyrosine proteome profiling of EHEC O157:H7 and *E. coli* K12 using gel-free mass spectrometry-based phosphoproteomics, which revealed a total of 512 unique sites from which five enriched phosphotyrosine site motifs were identified. We show that tyrosine phosphorylation targets proteins involved in various cellular processes and virulence. We demonstrate the functional importance of a tyrosine phosphorylated residue on the regulator SspA involved in the regulation of T3SS expression. By addressing the functional importance of *etk* and *wzc* for tyrosine phosphorylation and their effects on metabolism and virulence-related phenotypes, we demonstrate that the known tyrosine kinases Etk and Wzc do not explain the observed phenotype of the *etk wzc* double mutant. Thus, in addition to providing novel insights into phosphotyrosine-mediated regulation in bacteria, our findings strongly suggest the presence of hitherto unidentified tyrosine kinases in EHEC O157:H7.

## Results

### Extensive tyrosine phosphorylation of *E. coli* proteins

**Figure 1:**
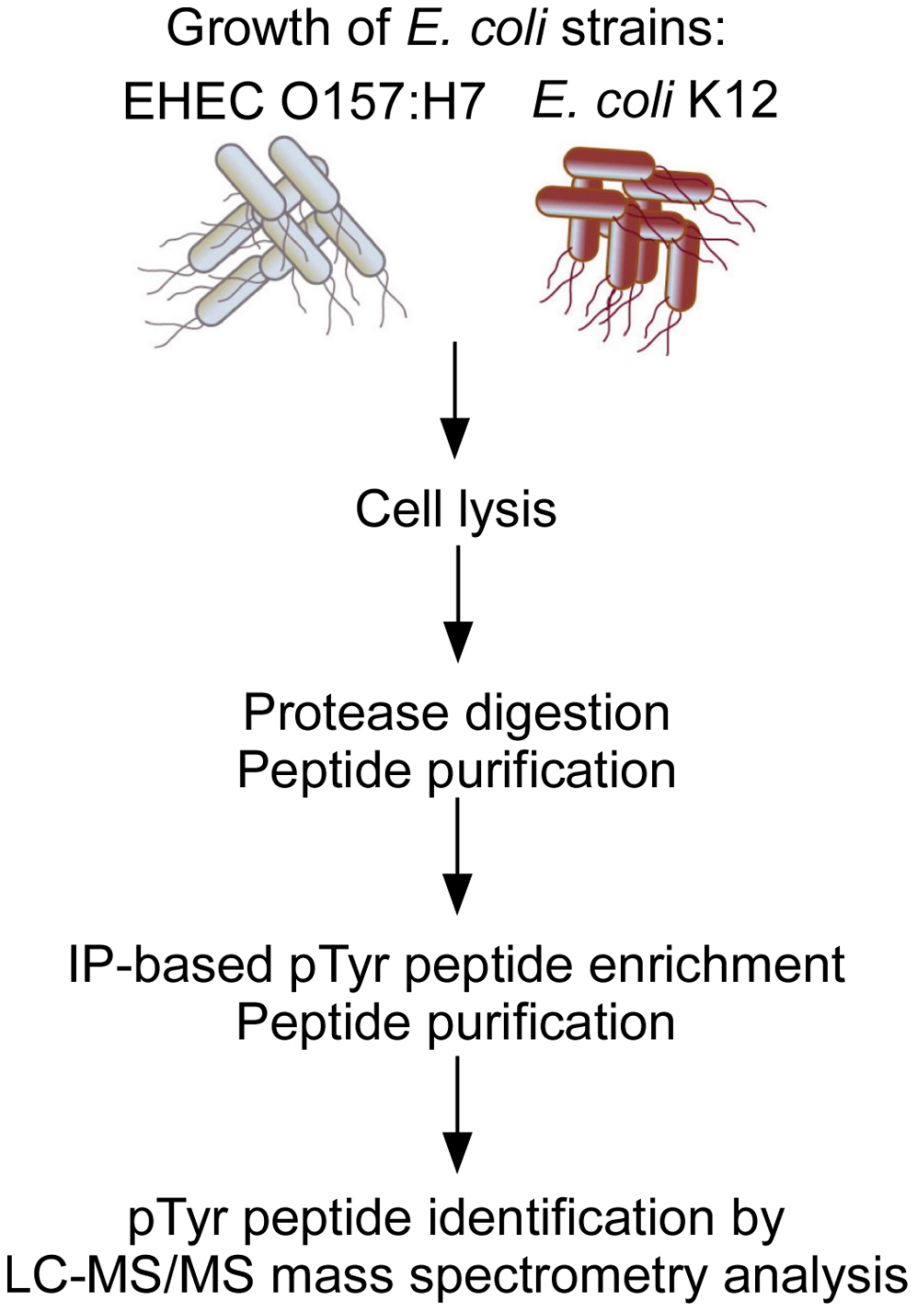
Proteomics approach used for phosphotyrosine profiling of *E. coli*. EHEC O157:H7 strain TUV93-0 and *E. coli* K12 strain MG1655 were grown to stationary phase in DMEM. Total protein was trypsin-digested, peptides purified using reversed-phased chromatography and subjected to immunoaffinity (IP)-based phosphotyrosine enrichment. Tyrosine phosphorylated peptides were identified by high resolution LC-MS/MS analysis using LTQ Orbitrap XL and Velos instruments.

We used a phosphoproteomic approach to comprehensively define the *E. coli* phosphotyrosine proteome and accurately map phosphorylation sites as outlined in figure 1. Protein extracts were prepared from cultures of *E. coli* K12 strain MG1655 [21] and EHEC O157:H7 strain TUV93-0, a Shiga toxin-deleted derivative of the strain EDL933 [8], [22], which was associated with an outbreak of hemorrhagic colitis in the United States in 1982 [23]. Strain TUV93-0 is hereafter referred to as wild type EHEC O157:H7. We enriched tyrosine phosphorylated peptides from trypsin-digested cell lysates using anti-pTyr antibodies followed by peptide identification using high-resolution liquid chromatography tandem mass spectrometry (LC-MS/MS) analysis on Linear Trap Quadrupole (LTQ)-Orbitrap mass spectrometers. The peptides were fragmented in both collision-induced dissociation (CID) and higher collision dissociation (HCD) modes to increase the confidence and coverage of identified peptides. Stringent data analysis parameters were applied to ensure confident identification of phosphotyrosine peptides. We identified 166 unique pTyr peptides containing 167 unique pTyr sites on 117 proteins from *E. coli* K12; and 561 unique pTyr peptides containing 416 unique pTyr sites on 287 proteins from EHEC O157:H7 with a false discovery rate of less than 1% (Table S1). Localization of phosphotyrosine sites assessed using PhosphoRS revealed probability scores of >95% for the majority of identified peptides, reflecting high accuracy of phosphotyrosine site assignments. The presence of diagnostic peaks corresponding to pTyr immonioum ions in MS/MS spectra obtained in the HCD fragmentation mode further confirmed identified phosphotyrosines (see figure S1 for representative MS/MS spectra). The increased number of phosphorylation events in EHEC O157:H7 could be attributed to increased activity of the tyrosine kinase Etk as reflected by a greater extent of phosphorylation of Tyr residues within the carboxyl-terminus Tyr cluster of Etk with 89 unique peptides detected in EHEC O157:H7 and only four in *E. coli* K12 (Table S1). This was also evident by higher levels of tyrosine phosphorylated Etk detected in EHEC O157:H7 by western blot analysis (Figure S2). Notably, the phosphotyrosine proteomes of EHEC O157:H7 and *E. coli* K12 represent two snapshots since these strains were grown under different conditions as described in *Material and Methods* to obtain increased coverage of the phosphotyrosine proteome. In all, specifically enriching for phosphotyrosine peptides rather than using the less specific metal affinity-based phosphopeptide enrichment approach used in most phosphoproteomic studies available for bacteria, combined with high resolution mass spectrometry allowed comprehensive identification of tyrosine phosphorylation events in *E. coli*. Our data revealed an *E. coli* phosphotyrosine proteome of 512 unique sites on 342 proteins, which is the highest number of tyrosine phosphorylated proteins reported in prokaryotes to date. Of 342 phosphotyrosine proteins, we were able to find published reports pertaining to only 18. Identified phosphotyrosine proteins comprise 4% and 6% of *E. coli* K12 and EHEC O157:H7 proteomes respectively, which exceeds tyrosine phosphorylation levels reported for mammalian cells [24].

### *E. coli* phosphotyrosine sites are conserved in related bacteria

To determine the conservation of the 512 unique phosphotyrosine sites identified in *E. coli*, we mapped sequences including five amino acid residues on each side of phosphorylated residues onto the proteomes of 16 evolutionary diverged bacteria. Conservation of *E. coli* phosphotyrosine sites was generally low (<10%) except for in evolutionary closely related bacteria *Salmonella enterica*, *Shigella flexneri*, *Klebsiella pneumoniae*, *Yersinia pestis*, *Vibrio cholerae* and *Haemophilus influenzae* (Table S2). With phosphotyrosine sites mainly being conserved in the enteric bacteria *E. coli*, *S. flexneri*, *S. entrica*, *V. cholerae* and *K. pneumoniae*, our data supports the idea that phospho-mediated regulation reflects adaptation to environmental niches [25]. Notably, at least two of four phosphotyrosine sites mapping to chaperone ClpB, translation elongation factor Tuf and ATP synthase subunit AtpD were present in all proteomes tested, reflecting early acquisition and potential functional importance of these sites. Indeed, the highly conserved ClpB Tyr653 residue identified in this study as phosphorylated was previously shown to be critical for substrate binding, and thereby chaperone function [26].

### Identification of phosphotyrosine site motifs

**Figure 2:**
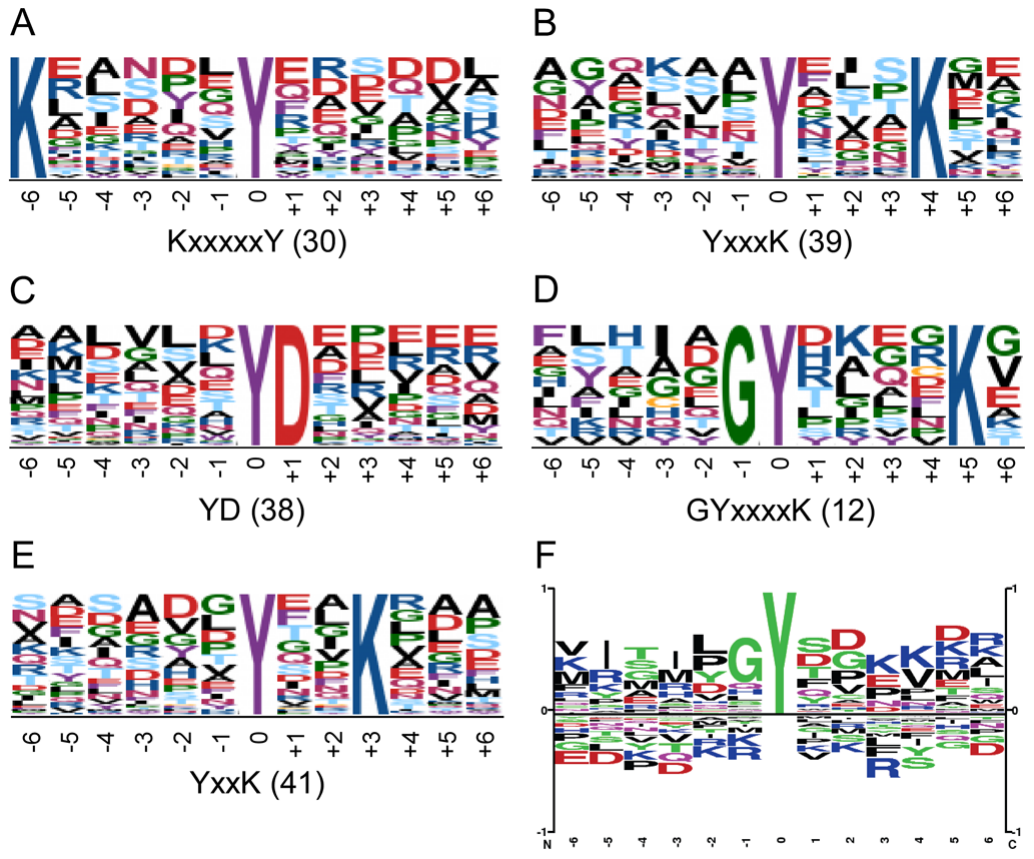
Definition of *E. coli* tyrosine phosphorylation site motifs. (A–E) Probability logos of significantly enriched phosphotyrosine site motifs extracted from 512 unique pTyr sites by aligning peptide sequences comprising 12 residues surrounding the phosphorylated tyrosine residue using Motif-X (*p value*<0.001). Site motif consensus sequences with variable residues indicated as x and the number of unique sites comprising each motif are indicated. (F) Sequence logos of the general residue representation surrounding the phosphorylated tyrosine residue from the 512 unique sites with residues above the midline being overrepresented and those below underrepresented constructed using Phophosite logo generator.

Whereas definition of tyrosine phosphorylation site motifs in bacteria has so far been limited by the low number of phosphotyrosine sites available, the extensive dataset of 512 unique pTyr sites identified here provided a reliable source for bioinformatics prediction of such motifs. We used the Motif-X algorithm [27] to extract enriched sequence motifs surrounding the identified phosphotyrosine sites by considering 12 residues centered on the phosphorylated tyrosine residue. We defined five statistically significantly enriched phosphotyrosine site motifs for 160 unique pTyr sites comprising 31% of sites identified (Figure 2A–E and Table S3). For four of these motifs, unambiguous enrichment of a positively charged lysine was observed at positions +3, +4, +5 and −6 relative to the phosphotyrosine respectively, suggesting that lysine might be functionally important for phosphorylation, potentially by acting as an electron sink. Moreover, glycine and aspartate were at positions −1 and +1 respectively; indeed, glycine at position −1 was also observed for phosphotyrosine sites in *Streptococcus pneumoniae* [28]. A general sequence motif derived from the 512 pTyr sites using Phosphosite logo generator [29] revealed significant overrepresentation of glycine and aspartate residues at positions −1 and +1 respectively, and underrepresentation of lysine residues at −1, −2, +1 and +2 (Figure 2F), which is consistent with the sequence composition of the five motifs identified.

To determine whether the five tyrosine phosphorylation site motifs from *E. coli* are prevalent in eukaryotes, we analyzed 7551 phosphotyrosine sites from human proteins catalogued in the Human Protein Reference Database [30] by considering 12 residues surrounding the phosphotyrosine using Motif-X. We identified 13 statistically significantly enriched sequence motifs: YSP, YxD, YDxP, YExP, YxSP,YxxxD, YxxxxK, DY, DxxY, ExxY, EDxY, SxxxY and RxxSxxY, where variable residues are indicated as x (*p value*<0.000001) (Figure S3 and Table S4). None of the human protein phosphotyrosine site motifs were identical to those of *E. coli*. However, one human protein pTyr site motif was similar to the *E. coli* motif GYxxxxK but lacks glycine at −1 (Figure S3), which might provide structural flexibility to the region of the tyrosine phosphoacceptor. The different composition of phosphotyrosine site motifs identified in human and bacterial proteins are consistent with the finding that BY kinases differ structurally from their eukaryotic counterparts [14], likely also implying different substrate specificities.

### Phosphotyrosine proteins are related to various cellular functions

**Figure 3:**
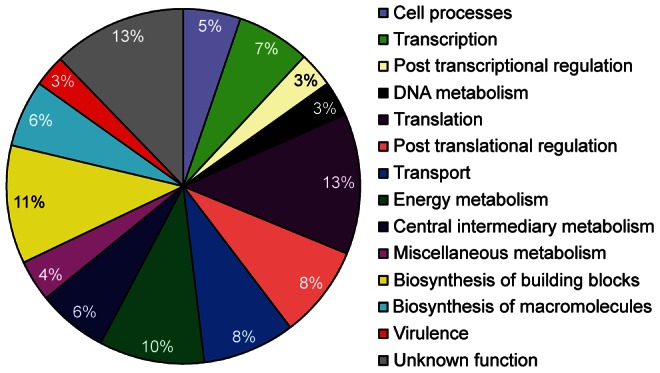
Phosphotyrosine proteins are involved in various cellular processes. Pie diagram depicting the functional classification of tyrosine phosphorylated proteins identified in *E. coli* according to biological processes as shown in table S5. The cell processes category includes cell division and stress adaptation.

Functional classification of the 342 phosphotyrosine proteins identified combined from EHEC O157:H7 and *E. coli* K12 revealed a broad range of fundamental cell processes including cell division, transport, transcriptional and translational levels of gene expression, various metabolic pathways, and virulence (Figure 3 and Table S5). The representation of proteins belonging to most functional classes is also evident in bacterial serine and threonine phosphoproteomes including that reported for *Mycobacterium tuberculosis* [3]. Functional group profiles of the two *E. coli* strains were similar despite a limited overlap of 60 proteins, which might be explained by the fact that the two strains were grown under different conditions as described in *Materials and Methods*, resulting in two unique snapshots of the *E. coli* phosphotyrosine proteome. Moreover, *E. coli* stains have an extensive mosaic genomic structure with only 64% of all proteins being shared between *E. coli* K12 and EHEC O157:H7 [31], which further could account for the differences in the phosphotyrosine protein profiles. Proteins identified as tyrosine phosphorylated confer important functions in their respective classes such as cell division (FtsA, FtsI, FtsK, FtsZ, YihA, MreB, MukB and ZipA), DNA synthesis (HolE) and DNA metabolism (GyrB, RecA, RecT, SbcB, Sms, TopA and Tus). Tyrosine phosphorylation is evident in translation, where proteins associated with initiation (InfA, InfB, InfC and YciH) and elongation (TufA, TufB and Tsf) were phosphorylated in addition to 27 ribosomal proteins. Our study identified phosphotyrosines on additional 21 ribosomal proteins compared to a previous proteomic analysis of ribosomal protein phosphorylation, where 12 proteins were identified as tyrosine phosphorylated [32]. Phosphotyrosine proteins implicated in protein turnover included proteases (ClpP, GlpG, HslU, Lon and Ptr) and chaperones (ClpB, DnaK, GroEL, GroS, HtpG and SurA). More than one-third of all phosphotyrosine proteins (36%) were associated with metabolic pathways, included those of energy- and central intermediary metabolism, biosynthesis of building blocks (amino acids, nucleotides, cofactors and fatty acids) and biosynthesis of macromolecules, most of which are cell surface exopolysaccharides. Moreover, multiple transport proteins including those of ATP-binding cassette family transporters and phosphotransferase systems were identified as tyrosine phosphorylated. Notably, proteins associated with metabolism, gene expression and virulence were tyrosine phosphorylated as discussed below. Another 42 phosphotyrosine proteins were of unknown function with the majority being hypothetical proteins whose expression was confirmed in this study. Overall, our data indicate a central role of tyrosine phosphorylation in most fundamental cellular processes. Identification of phosphotyrosine modifications on at least seven essential *E. coli* proteins (Frr, DnaK, FtsZ, InfA, Map, Ppa and ProS) further suggests a functional importance of tyrosine phosphorylation.

### Phosphotyrosine proteins are functionally enriched and central in metabolism

**Figure 4:**
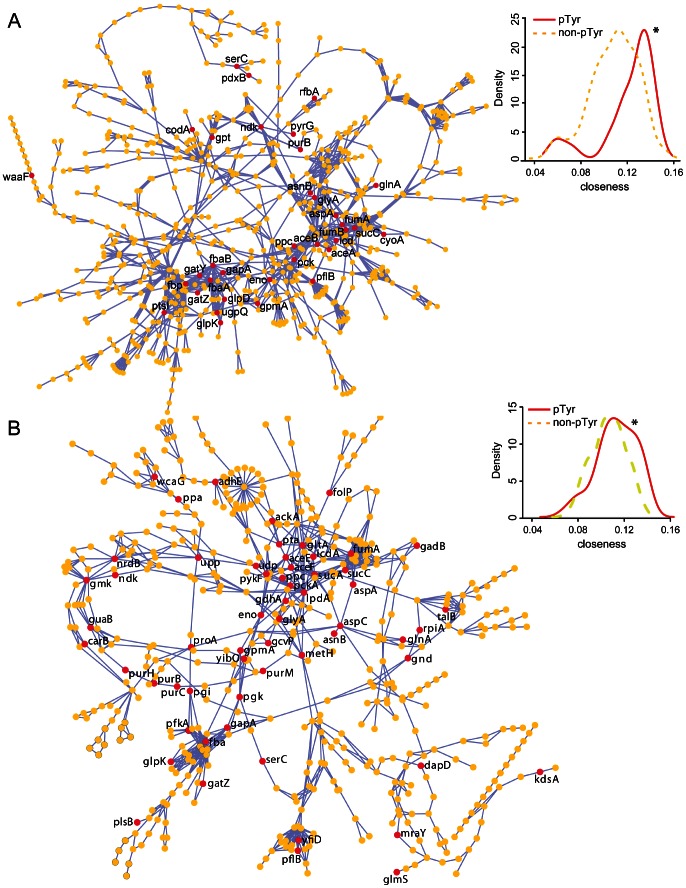
Phosphotyrosine proteins are central in the metabolic network. Protein-centric network representations of *E. coli* K12 (A) and EHEC O157:H7 (B) metabolism with pTyr (red nodes) and non-pTyr (yellow nodes) proteins indicated. Nodes represent proteins and edges represent compounds produced by one protein and consumed by other protein. Only names for the phosphotyrosine proteins are shown. Density plots showing the distribution of centrality closeness of pTyr (red) and non-pTyr (yellow) proteins in the metabolic network are shown. The higher the closeness centrality of a given protein (node), the shorter is its geodesic distances to other nodes in the graph.

Functional enrichment analysis was conducted to determine if phosphotyrosine proteins tend to participate in certain biological processes by contrasting the functional annotations of phosphotyrosine proteins against the total proteomes of EHEC O157:H7 EDL933 and *E. coli* K12 MG1655. We observed significant (*p value*≤0.05) enrichment of phosphotyrosine proteins in a wide range of metabolic and regulatory processes including the tricarboxylic acid cycle, glycolysis, gluconeogenesis, purine nucleotide biosynthesis pathways, and post-transcriptional regulation of gene expression (Table S6). Moreover, a graph-based analysis of the metabolic network revealed that tyrosine phosphorylated proteins are strikingly positioned centrally in this network (Figure 4), as they show significant higher closeness centrality values than non-pTyr proteins in the network for both *E. coli* K12 (*p value* = 2×10^−5^) and EHEC O157:H7 (*p value* = 1.5×10^−2^). Altogether, we demonstrated that phosphotyrosine proteins are enriched in various biological processes and are central in the metabolic network of *E. coli*.

### Tyrosine phosphorylation of proteins related to transcriptional and post-transcriptional control of gene expression

While phosphotyrosine modification is a well-established means to regulate eukaryotic transcription [33], [34], it is less common in prokaryotes. Recent phosphoproteomic studies revealed Ser/Thr phosphorylation of RNA polymerase (RNAP) core subunits [17], [28], whereas no phosphotyrosine events were observed. Here, we identified tyrosine phosphorylation of 22 transcription-related proteins including the RNAP core enzyme subunits α (RpoA), β (RpoB) and β′ (RpoC) (Table S5). Whether tyrosine phosphorylation of the bacterial RNAP affects the association with transcription factors as shown for yeast RNAP II [34] or RNAP assembly remains to be determined. Proteins affecting RNAP sigma factor availability and transcription elongation were also tyrosine phosphorylated, suggesting a role of phosphotyrosine-mediated signaling in controlling RNAP promoter specificity and activity. We detected tyrosine phosphorylation of transcriptional regulators of cysteine biosynthesis (CysB), nucleotide synthesis (PurR) and major carbon- and energy metabolic pathways (Cra), for which phosphorylated residues located within helix-turn-helix motifs potentially could affect DNA-binding activity as shown for sigma factor RpoH [35]. Also, tyrosine phosphorylation controls binding to DNA of a single-stranded DNA-binding protein in *Bacillus subtilis* [36]. Additional phosphotyrosine modified regulators include those involved in two-component response systems (OmpR, PhoP, RcsB and UvrY), multidrug resistance (EmrR), glucose-phosphate stress (SgrR), phosphate starvation- and anaerobiosis-associated stress (AppY), stationary phase-related stress (SspA), iron uptake (Fur), and nucleoid structure (Cnu, Dps, Hha, H-NS and IHF). The histone-like protein, H-NS, is a global modulator of stress response and virulence gene expression in bacterial pathogens including EHEC [37]. Interestingly, the phosphorylated Tyr61 and Tyr99 residues of H-NS are located in hydrophobic cores of the functionally important oligomerization and DNA-binding domains [38], [39], and could affect H-NS activity. Indeed, tyrosine phosphorylation determines the ability of eukaryotic histones to form protein associations and subsequently modulate gene expression [40]. Further evidence that tyrosine phosphorylation targets functionally important residues includes modification of the response regulator PhoP switch residue Tyr98 [41] and SspA Tyr92, which protrudes from a surface-exposed pocket and is critical for activity [42]. At least six of these transcription factors (Cra, Hha, H-NS, IHF, RcsB and SspA) are known to control gene expression from PAIs in EHEC [43]–[45].

Post-transcriptional regulation is known to be crucial for proper regulation of various fundamental bacterial cell processes, and has recently emerged as an important means to control virulence gene expression in various bacterial pathogens including EHEC O157:H7. We identified phosphotyrosine modifications of proteins involved in RNA metabolism comprising RNases (VacB and RNaseE), RNA chaperones (CsrA and ProQ) and a RNA helicase (RhlB) (Table S5), suggesting that tyrosine phosphorylation potentially also affects gene expression at the post-transcriptional level in *E. coli*. The expression of the LEE pathogenicity island is regulated post-transcriptionally by RNaseE directly and through Hfq-mediated small RNA regulation in EHEC [46], [47], and by CsrA in EPEC [48], suggesting that tyrosine phosphorylation might control virulence at the post-transcriptional level of gene expression. The possible involvement of phosphotyrosine modifications in post-transcriptional regulation in bacteria is supported by findings demonstrating a functional importance of tyrosine phosphorylation in eukaryotic RNA metabolism [19], [49]. Although an effect of tyrosine phosphorylation on the identified proteins remains to be directly demonstrated, our data reveal the potential for phosphotyrosine-mediated signaling in bacterial gene expression is much more widespread than previously known.

### Proteins expressed from EHEC O157:H7 pathogenicity islands are tyrosine phosphorylated

We identified 28 phosphotyrosine proteins expressed from EHEC O157:H7 PAIs (Table 1). Importantly, T3SS proteins including translocon components (EspA, EspB and EspD), intimin (Eae), chaperones (CesA and CesT), and the effectors EspJ and EspP were among the identified proteins. This is the first demonstration that proteins of a T3SS are tyrosine phosphorylated by bacterial kinases. The phosphorylated EspB residues, Tyr75 and Tyr272, are located in regions involved in the interaction with EspD [50], and might affect the association of these two pore-forming proteins. To assess the functional importance of these phosphotyrosine residues of EspB we created single and double mutants, which have the phosphorylated Tyr residues substituted with Phe, and tested their ability to complement A/E lesion formation of an *espB* mutant strain. The mutant EspB derivatives fully complemented the A/E lesion phenotype, (data not shown), suggesting that Tyr75 and Tyr272 are redundant for EspB activity *in vivo*. However, we cannot exclude the possibility that potential compensatory protein-protein interactions present between EspB and its target proteins account for the lack of an *in vivo* phenotype. It remains to be demonstrated whether tyrosine phosphorylation of any other T3SS proteins identified affects T3SS apparatus assembly and the activity of effectors transclocated into host cells.

**Table 1:** Identified phosphotyrosine proteins expressed from pathogenicity islands in EHEC O157:H7 listed as type III system (T3SS) and non-T3SS proteins.

Accession	Protein	Phosphorylation site(s)	Description
	T3SS		
NP_290286.1	CesA**	Y64	EspA chaperone
NP_290260.1	CesT	Y152/153	Tir chaperone
NP_290259.1	Eae	Y7	Intimin
NP_290256.1	EspA	Y192	Filament-forming translocated protein
NP_290254.1	EspB	Y75/272	Pore-forming translocated protein
NP_290255.1	EspD	Y137	Pore-forming translocated protein
NP_288436.1	EspJ	Y22/50	Translocated effector protein
NP_290286.1	Z5137	Y168	LEE1-encoded protein of unknown function
	Non-T3SS		
NP_290084.1	ChuX	Y86	Heme-binding protein
YP_325580.1	EspP	Y189	Type V-secreted serine protease
NP_286713.1	Iha	Y276	Bifunctional enterobactin receptor and adhesin protein
YP_325626.1	L7066	Y96	Hypothetical protein encoded by pO157
NP_288542.1	Per	Y7/218	Perosamine synthetase, O157 antigen synthesis
NP_286709.1	TerC	Y164	Phage inhibition, colicin resistance and tellurite resistance protein
NP_288541.1	WbdP	Y80/81/245/248/356	Glycosyl transferase, O157 antigen synthesis
NP_288545.1	Wzy	Y390	O157 antigen polymerase, O157 antigen synthesis
NP_285993.1	Z0342	Y172	Putative LysR-like transcriptional regulator
NP_286017.1	Z0367	Y85	Protein of unknown function
NP_286656.1	Z1121	Y204/291	Hypothetical protein
NP_287037.1	Z1533	Y201	Putative oxidoreductase
NP_287504.1	Z2040	Y69	Hypothetical protein
NP_287538.1	Z2080**	Y75	Putative IS-encoded protein in CP-933O
NP_287748.1	Z2309*	Y76	Hypothetical protein
NP_288200.1	Z2800*	Y68	Hypothetical protein
NP_288905.1	Z3594	Y57	Hypothetical protein
NP_289066.1	Z3776	Y4	Hypothetical protein
NP_289194.1	Z3939	Y56	Hypothetical protein
NP_290917.1	Z5897	Y458	Hypothetical protein

* Detected in the EHEC *wzc etk* double mutant.

** Detected in wild type EHEC from the experiment run in parallel with the EHEC *wzc etk* mutant sample.

We also identified phosphotyrosine modifications on non-T3SS virulence-associated proteins including the host cell adhesin Iha, and proteins related to tellurite resistance (TerC) and iron acquisition (ChuX). Due to limited iron availability in the host intestine, iron acquisition systems are important for EHEC to survive and successfully colonize the host. Interestingly, the phosphorylated residue of ChuX, Tyr86, is located in close vicinity to a putative heme-binding cleft [51], and therefore might affect heme uptake and stability. The involvement of tyrosine phosphorylation in extracellular polysaccharide synthesis, important for virulence *in vivo* [16], [52], was further implicated by the identification of at least 13 phosphotyrosine proteins related to lipopolysaccharide synthesis of which four are involved in O157 antigen synthesis (Glf, Per, WbdP and Wzy). About half of the identified PAI-encoded phosphotyrosine proteins are of unknown function but could potentially also be implicated in virulence, given the location of their genes on pathogenicity islands. Our data suggests that phosphotyrosine modifications by BY kinases in addition to host tyrosine kinases could play a central role in controlling EHEC O157:H7 virulence.

### Mutation of the phosphotyrosine site in SspA negatively affects virulence of EHEC O157:H7

**Figure 5:**
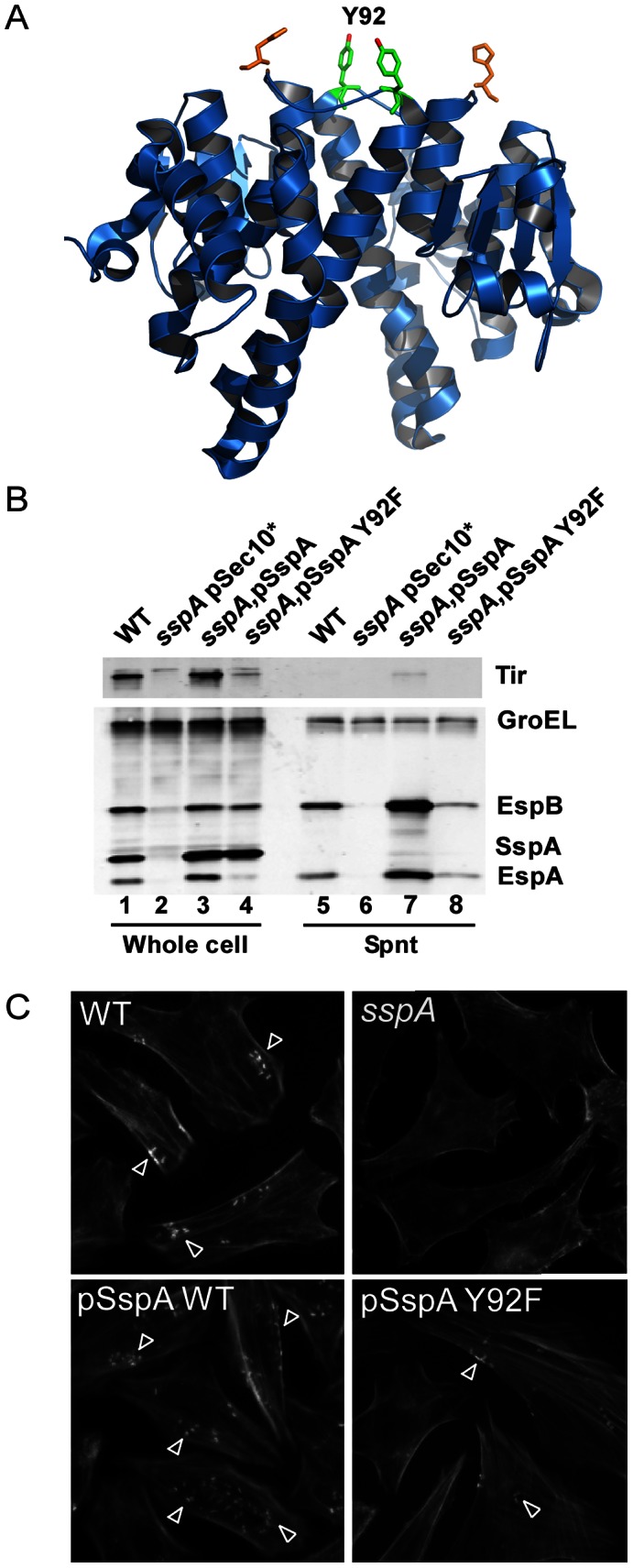
Tyrosine phosphorylation of SspA Tyr92 affects virulence phenotypes of EHEC O157:H7. (A) Location of Tyr92 in dimeric SspA (PDB 1YY7 [42]). The structure of dimeric SspA is shown as blue ribbon diagrams. The hydrophobic residues Tyr92 and His85 of the functionally important surface-exposed pocket are shown in green and orange, respectively. The hydroxyl group of Tyr92 that is subject to phosphorylation is shown in red. The SspA structure was visualized using PyMOL (Schrödinger LLC). (B) SspA Tyr92 positively affects expression and secretion of T3SS proteins. The abundance of LEE-encoded proteins in whole cell lysates (lanes 1–4) and their abundance in culture supernatants (lanes 5–8) from cultures of wild type EHEC O157:H7 and isogenic *sspA* mutants were determined by western analyses as described in *Material and Methods*. Strains tested included the *sspA* mutant containing the vector control pSec10*, the *sspA* mutant expressing wild type SspA from pSspA and the SspA Y92F mutant from pSspAY92F. EspA, EspB, Tir, SspA and GroEL were detected using polyclonal antisera against the respective proteins. GroEL served as an internal control for the total amount of protein in cell samples, and for the precipitation of proteins in culture supernatants to which 100 ng of GroEL were added. (C) SspA Tyr92 positively affects the A/E phenotype of EHEC O157:H7. A/E lesion formation was assessed using the FAS test as described in *Material and Methods*. HeLa cells were co-cultured for 5 h with wild type EHEC O157:H7, an *sspA* mutant and the *sspA* mutant harboring the vector pSec10*, pSspA (SspA) and pSspAY92F (SspAY92F). The actin cytoskeleton of HeLa cells was stained with FITC-phalloidin for visualization of the A/E lesions. Representative images of fluorescence stained actin of infected HeLa cells are shown. Arrows indicate examples of A/E lesions.

To directly demonstrate the role of tyrosine phosphorylation in the function of SspA, which is part of the complex regulatory network controlling expression of the T3SS in *E. coli* [43], [45], we mutated the SspA Tyr92 residue identified here as phosphorylated. SspA positively affects the expression of the H-NS regulon including many virulence-associated proteins by negatively controlling H-NS levels [45], [53]. A surface-exposed pocket of SspA that includes the Tyr92 residue is important for SspA activity [42] (Figure 5A), raising the question of whether phosphorylation of Tyr92 affects SspA activity. To assess the functional importance of Tyr92 phosphorylation we replaced this residue with Phe, which is non-phosphorylatable due to the lack of the hydroxyl group of Tyr that is target for phosphorylation (SspA Y92F). Deletion of the *sspA* gene results in loss of T3SS-associated proteins and A/E lesion formation (Figure 5B and C). We compared the ability of wild type SspA and the mutant SspA Y92F encoded on plasmids along with the empty plasmid vector to complement the defect of a *sspA* mutant in these phenotypes. The SspA Y92F mutant exhibited decreased ability to complement the expression of the LEE-encoded proteins EspA, EspB and Tir compared to wild type SspA (Figure 5B, compare lanes 3 and 4). Moreover, we observed decreased abundance of these T3SS-associated proteins in culture supernatants from the *sspA* mutant strain expressing SspA Y92F (Figure 5B, compare lanes 7 and 8). We tested the ability of SspA Y92F to support a *sspA* mutant in A/E lesion formation on HeLa cells using fluorescence actin staining assay (FAS), where lesions are visualized as condensed FITC-phalloidin stained actin beneath adherent bacteria [54]. The decreased abundance of T3SS-associated proteins was reflected by a less pronounced A/E lesion phenotype when complementing the *sspA* mutant with SspA Y92F compared to wild type SspA (Figure 5C, lower panels), indicating functional importance of the phosphorylated SspA residue Tyr92 in regulating expression of virulence-associated proteins in EHEC O157:H7. To further verify the functional role of Tyr92 phosphorylation in SspA activity, we constructed phosphomimetic mutants with Tyr92 replaced with Asp (SspA Y92D) and Glu (SspA Y92E), and tested their ability to complement LEE expression and secretion of LEE-encoded proteins in a *sspA* mutant by western analyses (Figure S4). We observed partial restoration of LEE expression and protein secretion in the *sspA* mutant when complementing with the phosphomimetic SspA mutants to a degree higher than the non-phosphorylatable SspA Y92F mutant (Figure S4, compare lanes 5–6 with lane 4; and lanes 11–12 with 10) but to a lower level than wild type SspA (Figure S4, compare lanes 5–6 with lane 3; and lanes 11–12 with lane 9). The SspA Y92D mutant seemed to be more effective than the SspA Y92E mutant in mimicking the effect of a phosphotyrosine (Figure S4, compare lanes 5 and 11 with lanes and 6 and 12). The partial complementation of SspA activity by phosphomimetic SspA could be due to the possibility that the Asp and Glu do not completely reproduce the effect of Tyr92 phosphorylation structurally and electrostatically. Strikingly, a multiple sequence alignment revealed that 15 of 50 SspA orthologs contain either Asp or Glu at position 92 instead of Tyr [42], which further emphasizes the importance of a negative charge at SspA position 92 for activity whether it is permanently present as Asp/Glu residues or introduced by phosphorylation of Tyr as a mean to regulate SspA activity. In sum, our results strongly indicate that phosphorylation of SspA Tyr92 regulates SspA activity, and thereby virulence of EHEC O157:H7.

### Deletion of *etk* and *wzc* does not alter virulence-related and metabolic phenotypes in EHEC O157:H7

**Figure 6:**
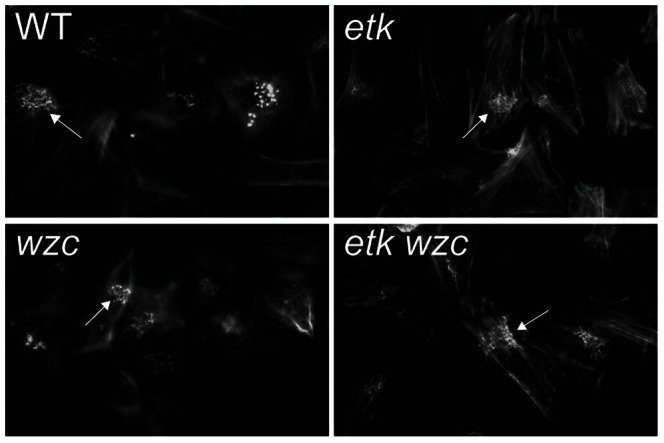
BY kinases Etk and Wzc are dispensable for the A/E lesion phenotype of EHEC O157:H7. HeLa cells were co-cultured with wild type EHEC O157:H7, *etk*, *wzc* and *etk wzc* double mutant derivatives for 5 h followed by actin staining of infected cells using FITC-phalloidin to visualize A/E lesion formation. Representative images of FITC-phalloidin stained actin of infected HeLa cells are shown with arrowheads indicating A/E lesions.

To address the functional importance of the two currently known BY kinases, Etk and Wzc, in metabolism and virulence, we generated deletion mutants of *etk* and *wzc* in EHEC O157:H7. Since T3SS-associated virulence factors were among identified phosphotyrosine proteins, we tested the ability of wild type and the tyrosine kinase mutants to form A/E lesions on HeLa cells using the FAS assay. Similar A/E lesion phenotypes were observed for wild type, *etk*, *wzc* and *etk wzc* mutant strains (Figure 6), indicating that Etk and Wzc are dispensable for pedestal formation. Consistent with comparable A/E lesion phenotypes, western blot analysis revealed similar levels of the T3SS proteins EspA and EspB in cell lysates and culture supernatants of *etk* and *wzc* mutant derivates and wild type (Figure S5A). Though an EHEC O157:H7 *etk* mutant was previously shown to exhibit two-fold increased pedestal formation efficiency [52], we did not observe any obvious effects of deleting *etk* or *wzc* in our assay. Our data suggest that tyrosine phosphorylation of T3SS-associated virulence proteins by Etk and/or Wzc either is not functionally important for A/E lesion formation, or else is mediated by other tyrosine kinases.

Since the major functional class of tyrosine phosphorylated proteins involves metabolic pathways, we next determined the role of Etk and Wzc in *E. coli* metabolism by comparing the ability of EHEC O157:H7 wild type and *etk wzc* double mutant strains to metabolize various carbon, nitrogen, phosphorus and sulfur sources using Biolog Phenotype Microarrays [55]. Surprisingly, the metabolic profiles of wild type and *etk wzc* double mutant strains were similar (Figure S5B), indicating that tyrosine phosphorylation by Etk and Wzc is dispensable for metabolizing these analytes.

### Deletion of known tyrosine kinases does not abolish tyrosine phosphorylation in EHEC O157:H7

The absence of an effect on metabolism and virulence of EHEC O157:H7 when deleting *etk* and *wzc* despite the extensive tyrosine phosphorylation observed for proteins involved in these processes suggested that hitherto unidentified tyrosine kinases exist in *E. coli* to functionally compensate for these kinases. Indeed, such functional redundancy has been reported for Src family tyrosine kinases in eukaryotes [56]. To address this possibility, we carried out a second set of phosphotyrosine profiling experiments, where samples of EHEC O157:H7 wild type and *etk wzc* double mutant strains were run in parallel using identical experimental conditions for qualitative comparison. Interestingly, the *etk wzc* double mutant showed residual tyrosine phosphorylation with 115 unique pTyr peptides comprising 103 unique sites identified on 81 proteins, while 279 unique pTyr peptides comprising 237 unique sites on 168 proteins were identified in the wild type (Table S1). Interestingly, nearly all (93) of the 103 unique pTyr sites identified in the *etk wzc* double mutant were also present in the phosphotyrosine proteome of wild type EHEC O157:H7 defined in the first profiling experiment of this study. Our findings strongly indicate that additional tyrosine kinase(s) in *E. coli* provide at least a partial functional redundancy for tyrosine phosphorylation by Etk and Wzc. 37% of the phosphotyrosine sites from the *etk wzc* double mutant could be assigned to one of the five pTyr site motifs extracted from wild type *E. coli* (Table S3), suggesting that (an) unknown tyrosine kinase(s) possess site specificity that overlaps with Etk and/or Wzc, similar to overlap that is common for serine/threonine kinases [3]. Identified phosphotyrosine proteins of the *etk wzc* double mutant represent all functional classes described for those identified in wild type including various metabolic pathways, gene expression and virulence. Among the phosphotyrosine proteins identified in the *etk wzc* double mutant strain were virulence-associated proteins such as the LEE-encoded EspA and CesT, which are part of the T3SS system, as well as ChuX and WbdP involved in iron acquisition and O157 antigen synthesis respectively (Table S1). Also, the global regulators H-NS and CsrA important for virulence of A/E pathogens were tyrosine phosphorylated in the *etk wzc* double mutant. Thus, functional redundancy of *E. coli* tyrosine kinases could in part explain the lack of associated metabolic and virulence-related phenotypes observed for an *etk wzc* double mutant.

The peptide identifications from the EHEC O157:H7 sample included in this second profiling experiment as a positive control for the detection of tyrosine phosphorylation revealed that tyrosine phosphorylation occurs reproducibly at specific sites. This reproducibility was reflected by the finding that 232 of 237 sites identified in the wild type EHEC O157:H7 sample in this second experiment were among the 416 unique sites identified in the EHEC O157:H7 sample analyzed in the first profiling experiment. It is known that repetitive LC-MS/MS runs of a given sample increase the number of peptide identifications resulting in increased proteome coverage [57]. Thus, the decreased number of phosphotyrosine sites identified in this second wild type EHEC sample (237) compared to that of that of the first experiment (416) could be due to the fact that these samples were analyzed by respectively three and ten LC-MS/MS runs.

### *In silico* search for BY kinase candidates in *E. coli*

Rather than sharing structural similarity to their eukaryotic counterparts most classical BY kinases contain conserved Walker A, and B motifs of P-loop containing nucleotide triphosphate hydrolases and a Walker A′ motif, defining the active site of the catalytic domain, followed by a carboxyl-terminus tyrosine cluster [58]. BY-kinases of *Proteobacteria* undergo a two-step activation process involving phosphorylation of the catalytic residue Tyr574 of Etk followed by salt bridge formation with Arg617 and autophosphorylation of the carboxyl-terminus tyrosine cluster [59], [60]. Structure-based *in silico* sequence analyses involving similarity searches of BY kinase catalytic domains were used to predict tyrosine kinase candidates. PSI-BLAST searches of catalytic domain residues of Etk and Wzc against EHEC O157:H7 and *E. coli* K12 total proteomes revealed similarity to the plasmid partition protein SopA (Table S7). Moreover, fold recognition analyses revealed the fold of nucleotide-binding proteins characteristic for Etk and Wzc in the cell division ATPase MinD and the plasmid partition proteins ParA and SopA (Table S8). This is consistent with previous *in silico* analyses revealing sequence significant similarity of BY kinases to MinD and ParA family proteins [58]. MinD lacks tyrosine kinase activity [61] and ParA is absent from EHEC EDL933, and therefore cannot explain the residual tyrosine phosphorylation observed in the *etk wzc* double mutant. Structure-based sequence alignment of a ParA-based SopA model (Figure S6A) and Etk revealed conservation of Walker motifs A, A′ and B. However, the catalytic residues Tyr574 and Arg614 of Etk were substituted with Thr149 and Ser196, respectively, in SopA (Figure S6B). Potentially, a compensatory interaction between SopA residues Tyr153 and Arg346, located within a 4 Å range, might mimic that of Tyr574 and Arg614 in Etk (Figure S6C). However, SopA was not among the identified tyrosine phosphorylated proteins, which argues against SopA being a tyrosine kinase since the activity of classical BY kinases usually requires autophosphorylation. Thus, definitive identification of SopA as a tyrosine kinase will require further functional studies which are beyond the scope of this study.

## Discussion

Although tyrosine phosphorylation has become a recognized protein modification in bacteria, the extent of this modification remains uncertain. Using a combined immunoaffinity enrichment and mass spectrometry-based phosphoproteomic approach we established tyrosine phosphorylation in bacteria as far more prevalent than previously known with 512 unique pTyr sites identified on 342 proteins from *E. coli*. Our results indicate that tyrosine phosphorylation targets a broad range of fundamental cellular processes ranging from control of gene expression to metabolic pathways, a range that is indicative of a global regulatory network that likely affects various aspects of bacterial cell physiology and virulence. In *E. coli* phosphotyrosine-based regulation appears highly integrated as reflected by our identification of phosphorylated proteins involved in the synthesis, turnover and modification of DNA, RNA and proteins. The complexity of phosphotyrosine-mediated signaling at multiple levels of regulation is exemplified by the identification of tyrosine phosphorylation of Cra, a global transcription regulator controlling the expression of enzymes involved in major carbon and energy metabolism pathways [62], as well as more than half of the Cra-controlled enzymes involved in these metabolic processes. Additional phosphotyrosine profiling studies of cells grown under different conditions could help unveil the biological role of tyrosine phosphorylation, such as whether, it like acetylation in *Salmonella enterica*, coordinates carbon source utilization and metabolic flux of the central carbon metabolism [63].

Tyrosine phosphorylation often affects protein activity and interaction properties. Indeed, several identified phosphotyrosine proteins are components of macromolecular complexes such as the transcription and translation apparatus, RNA degradosome, transporters and the T3SS machinery, likely implying a role of phosphotyrosine modifications in protein associations. Moreover, steric and charge effects caused by tyrosine phosphorylation could potentially modulate ligand binding of the ATP and metal ion-binding proteins. In fact, the chaperone ClpB Tyr503 residue, identified here as phosphorylated, is critical for ATP-binding [26]. We detected phosphorylation of many tyrosine residues previously reported as functionally important by other investigators, suggesting a central role of tyrosine phosphorylation in protein function. A functionally important surface pocket residue of the transcription regulator SspA (Tyr92) was among those identified as phosphorylated. We demonstrated that Tyr92 is involved in phosphotyrosine-mediated regulation of SspA since changing this residue to Phe decreased the ability of SspA to induce expression of T3SS proteins, which also was reflected by reduced A/E lesion formation (Figure 5). Moreover, the phosphomimetic mutants SspA Y92D and SspA Y92E, in contrast to the SspA Y92F mutant, positively affected LEE expression of a *sspA* mutant strain (Figure S4). Since SspA Tyr92 is important for SspA regulation of H-NS levels, phosphotyrosine-mediated regulation of SspA could very likely also affect stress responses important for virulence such as acid resistance. Further studies addressing the functional role of phosphotyrosine modifications are required to reveal an effect on other proteins identified in this study.

Functional classes of phosphotyrosine proteins involving transcription, translation and central metabolism exhibit a striking overlap with those of Ser/Thr phosphorylated [18], [28] and lysine acetylated proteins [64], [65], raising the possibility of crosstalk between different types of protein modifications. Such crosstalk is common in eukaryotes and was recently reported for *M. pneumoniae* [66]. For instance, proteins such as RNAP subunits and translation elongation factor EF-G that we identified as being tyrosine phosphorylated are also Ser/Thr phosphorylated and lysine acetylated [18], [28], [64], [65], which could affect phosphotyrosine-mediated regulation of these proteins. Indeed, the *Yersinia* T3SS effector YopJ acetylates Ser/Thr residues on target proteins, thereby blocking their phosphorylation [67]. Multiple central metabolic pathway enzymes identified here as tyrosine phosphorylated are also Ser/Thr phosphorylated and acetylated [18], [28], [63], suggesting the concerted action of these protein modifications in regulating metabolism. Ribosomal proteins can be Ser/Thr/Tyr phosphorylated at multiple sites (Table S1, [32]), probably affecting ribosome assembly and activity. Moreover, phosphotyrosine modification of translation initiation and elongation factors identified in this study along with Ser/Thr phosphorylation of these proteins reported in other studies [18], [28] further suggests that phospho-mediated signaling is central in regulating prokaryotic translation as reported for eukaryotes [68]. Thus, crosstalk between different types of protein modifications likely adds complexity to phosphotyrosine-mediated regulation, an already dynamic regulatory process, through the concerted actions of tyrosine kinases and phosphatases.

Our data indicate the presence of an as-yet undiscovered tyrosine kinase(s) in *E. coli* since tyrosine phosphorylation was present in the *etk wzc* kinase double mutant, thereby adding further complexity to the *E. coli* tyrosine kinome. A structure-based *in silico* search for additional BY kinases harboring Walker motifs revealed structurally highly similar P-loop NTPase family proteins including SopA for which tyrosine kinase activity has yet to be demonstrated. Though SopA could be a third kinase, additional hitherto unknown tyrosine kinases likely exist as the A/E lesion phenotype is intact when deleting *sopA* in the *etk wzc* tyrosine kinase double mutant of EHEC (data not shown). Notably, the ribosome-associated GTPase BipA that autophosphorylates on tyrosine positively affects pedestal formation by affecting LEE expression in EPEC [69], [70]. This could explain the phosphotyrosine modifications of T3SS proteins, although endogenous tyrosine phosphorylation by BipA has yet to be demonstrated. Although tyrosine phosphorylation in bacteria primarily is conducted by classical BY kinases, tyrosine kinases devoid of the canonical Walker motifs including eukaryotic-like and novel BY kinases [71], [72] could account for tyrosine phosphorylation observed in the absence of Etk and Wzc. Indeed, the Shiga toxin-encoding 933W prophage of EHEC O157:H7 strain EDL933 expresses a eukaryote-like tyrosine kinase, Stk, which is activated upon phage HK97 infection and is involved in phage exclusion [73]. However, Stk exhibits limited if any activity in uninfected cells and is absent from *E. coli* K12, thus being unlikely to account for the phosphotyrosine modification events detected in our study. Also, the A/E lesion phenotype of the EDL933 derivative used in this study was unaffected by the absence of *stk* in the *etk wzc* kinase double mutant (data not shown), suggesting that Stk along with Etk and Wzc is functionally redundant for virulence. Besides identifying additional BY kinases, further insight into environmental signals controlling the activity of kinases and cognate phosphatases is a prerequisite for understanding the regulatory effect of phosphotyrosine-mediated signaling in bacteria.

Tyrosine phosphorylation-dependent capsule synthesis is important for survival of EHEC O157:H7 and *Klebsiella pneumoniae* during infection [17], [52], currently providing the strongest link between BY kinases and virulence. An EHEC O157:H7 *etk* mutant devoid of group 4 capsule (G4C) was attenuated in an infant rabbit model [52], suggesting that G4C protects the pathogen from the host immune system, or that tyrosine phosphorylation by Etk positively affects additional factors required for colonization. Although the link of Etk to EHEC virulence via capsule formation was previously demonstrated, tyrosine phosphorylation of proteins that are encoded by EHEC O157:H7 PAIs, and thereby likely to be associated with virulence, had yet to be detected prior to this study. In this study, we identified numerous proteins encoded by PAIs as tyrosine phosphorylated such as those involved in O157 antigen synthesis, tellurite resistance, adhesion and iron acquisition. Most notably, we demonstrated that T3SS proteins including translocon components, chaperones and effectors are tyrosine phosphorylated by bacterial kinases. However, it remains to be determined whether tyrosine phosphorylation of T3SS proteins is required for secretion apparatus assembly and regulation of protein secretion as reported for Thr phosphorylation of type VI secretion system proteins in *Pseudomonas aeruginosa* [74]. Tyrosine phosphorylation by BY kinases could also affect the activity of the effectors EspG and EspP in host cells. Evaluation of this possibility requires experiments testing whether tyrosine to phenylalanine substitutions of the T3SS protein tyrosine residues detected as phosphorylated can affect T3SS function. Identified tyrosine phosphorylated proteins encoded by the *E. coli* K12 chromosomal backbone such as Cra, CsrA, H-NS, IHF and SspA further contribute to pathogenesis by controlling virulence- and stress response gene expression, thereby promoting survival of the pathogen in host environments. Indeed, we demonstrated functional importance in virulence of the phosphotyrosine residue of SspA. Taken together, our data suggests that phosphotyrosine modifications by bacterial kinases likely play a role in controlling various aspects of EHEC virulence.

In conclusion, we demonstrated extensive tyrosine phosphorylation of *E. coli* proteins involved in a wide variety of cellular processes, strongly suggesting that phosphotyrosine-mediated signaling coordinately regulates major cellular functions, and is thereby likely to be pivotal in controlling *E. coli* physiology and virulence. Proteins of a T3SS are here shown for the first time as being tyrosine phosphorylated by bacterial kinases. Importantly, our data demonstrate a functional role of phosphotyrosine-mediated regulation in controlling the expression of T3SS proteins associated with virulence. We defined five enriched phosphotyrosine site motifs and provided evidence for the presence of additional tyrosine kinases in *E. coli*. Altogether, our data provides a strong basis for further studies addressing the regulatory effect of tyrosine phosphorylation in *E. coli*.

## Materials and Methods

### Bacterial strain and plasmid construction

#### Strain constructions

Deletion mutant derivatives of *sspA*, *etk* and *wzc* were constructed by Lambda Red-mediated recombination using linear DNA fragments as described [75] in TUV93-0, a derivative of EHEC O157:H7 strain EDL933 that is deleted for Shiga toxin [22]. Briefly, DNA fragments encoding a kanamycin resistance cassette flanked by Flipase Recognition Target (FRT) site-inverted repeats and sequences homologous to regions flanking *sspA*, *etk* and *wzc* respectively, were PCR-amplified from pKD13 using primer sets AH1166/AH1167, K6486/K6487 and K6490/K6491 by the high-fidelity DNA polymerase EasyA (Agilent) (Oligo sequences are listed in table S9). The DNA fragments were electroporated into TUV93-0, where the Red recombinase system was expressed from pKD46. Recombinants were selected and purified on Luria Bertani (LB) agar plates containing 50 µg/ml kanamycin. The kanamycin resistance marker was eliminated from TUV93-0 Δ*sspA*::*kan*, TUV93-0 Δ*etk*::*kan* and TUV93-0 Δ*wzc*::*kan* using a pCP20-encoded FLP flipase [75]. The strain deleted for *etk* (TUV93-0 Δ*etk*::FRT, AMH130) has 2360 nt deleted starting from the position of the translation initiation codon, and is proceeded by an 83 nt long scar sequence originating from excision of the *kan* resistance marker. The strain deleted for *wzc* (TUV93-0 Δ*wzc*::FRT, AMH132) has ∼2067 nt deleted starting from position 126 nt within the coding region of *wzc* to avoid interruption of the overlapping gene *wzb*, and is proceeded by an 83 nt long scar sequence originating from excision of the *kan* resistance marker. An EHEC *wzc etk* double mutant strain (AMH136) was generated by deleting *etk* in strain AMH132 as described above. The *sspA* mutant strain contained an in-frame deletion allowing expression of the downstream gene *sspB* (TUV93-0 Δ*sspA*::FRT, AMH168). Gene deletions of *sspA*, *etk* and *wzc* were verified by DNA sequencing and PCR amplification analysis using primer sets AH1168/AH1169, K6488/K6489 and K6492/K6493 respectively.

#### Plasmid constructions

Oligo sequences used for plasmid constructions are listed in table S9.

pSspA (pAMH251): A ∼1.1 kb DNA fragment encoding *sspA* and ∼420 bp of the upstream regulatory region was PCR amplified from TUV93-0 gDNA by the EasyA DNA polymerase (Agilent) using oligos AH1168/AH1169, digested with *Sal*I and *Hind*III, and cloned into the corresponding sites of the low copy number vector pSec10 [76].

pSspAY92F(pAMH252): Site directed mutagenesis of *sspA* on pAMH251 resulting in the substitution Y92F was carried out using primer set AH1171/AH1172 and the QuickChange XL Site-Directed Mutagenesis Kit (Agilent) as recommended by the manufacturer.

pSec10*: pSec10 [76] was digested with *Hind*III and religated resulting in a pSec10 derivative with parts of the *clyA* gene deleted. This construct was used as a vector control.

### Phosphotyrosine profiling

#### *E. coli* cultures

Strains subject to phosphotyrosine profiling included *E. coli* K12 strain MG1655 [21], EHEC O157:H7 strain TUV93-0 [22], and a TUV93-0 *etk wzc* double mutant. EHEC O157:H7 and *E. coli* K12 strains were grown to stationary phase at 37°C under the following conditions. For EHEC O157:H7, overnight cultures of wild type TUV93-0 and *etk wzc* double mutant strains grown in LB medium were harvested, resuspended in an equal volume of 1× phosphate buffered saline (PBS), then diluted 1∶100 in Dulbecco's Modified Eagle Medium (DMEM, Invitrogen) and grown to an optical density value at 600 nm (OD_600_) of 1.0. For *E. coli* K12, MG1655 was grown overnight in 2 L of LB, cells were harvested, resuspended in an equal volume of DMEM and grown for additional 4 hours. Cells were harvested by centrifugation and washed twice with 1× PBS and stored at −80°C until use.

#### Phosphotyrosine peptide enrichment

Enrichment of tyrosine phosphorylated peptides from cell lysates was carried out using Phosphoscan Kit (P-Tyr-100, Cell Signaling Technology) as per the manufacturer's instructions with minor modifications. Briefly, 3 g wet weight of cell pellet were resuspended in 10 ml urea-containing buffer (20 mM HEPES pH 8.0, 9 M urea, 1 mM sodium orthovanadate, 2.5 mM sodium pyrophosphate, 1 mM ß-glycerophosphate) and sonicated using a microtip at a duty cycle of 40% and output control at 4 (Sonifier 250, Branson). Cell lysates were cleared by centrifugation at 20,000× *g* for 15 min at 15°C, reduced using 5 mM dithiotheitol at 60°C for 20 minutes. Cooled samples were subsequently treated with 20 mM iodoacetamide for 15 minutes in the dark. The lysates were diluted 4-fold to a final concentration of 2 M urea and 20 mM HEPES buffer (pH 8.0) and 30 mg protein were digested with 100 µg trypsin-TPCK overnight at room temperature with constant rocking. Lysate peptides were purified using a Sep-Pak C_18_ stage-tip (3M Empore) followed by extensive lyophilization. Phosphotyrosine peptides were enriched using pY-100 (Cell Signaling Technology) and 4G10 (Upstate Biotechnology) phosphotyrosine specific antibodies. Enriched peptides were purified using the Sep-Pak C_18_ stage-tips and analyzed by LC-MS/MS using two Fourier Transform mass spectrometers.

#### Mass spectrometry-based phosphotyrosine peptide identification

A total of 29 phosphotyrosine peptide samples were analyzed using Fourier Transform LTQ Orbitrap XL and Velos mass spectrometers (Thermo Fisher Scientific). The LTQ Orbitrap XL mass spectrometer was equipped with a nanoflow electrospray system connected to Eksigent nanoliquid chromatography and an Agilent 1100 microwell plate autosampler. The samples were analyzed in collision-induced dissociation (CID) mode, where MS data was acquired at a resolution of 60,000 at *m/z* 400 and MS/MS data was acquired in LTQ. The dried peptide samples were rapidly enriched (5 µl/min) on a trap column (75 µm×2 cm, Magic C_18_ AQ Michrom Bioresources, 5 µm, 100 Å) and separated on an analytical column (75 µm×10 cm, Magic C_18_ AQ Michrom Bioresources, 5 µm, 100 Å) at a flow rate of 300 nl/min. The samples were also analyzed on a LTQ Orbitrap Velos mass spectrometer interfaced with Agilent 1200 LC system in higher collision dissociation (HCD) mode. The MS and MS/MS data were acquired at a resolution of 60,000 at *m/z* 400 and 7,500 at *m/z* 400 respectively. For each cycle of data-dependent analysis 10 and 20 most abundant peptides were selected for MS/MS analysis in LTQ Orbitrap XL (normalized collision energy 37%) and LTQ Orbitrap Velos (normalized collision energy 40%) respectively. In the first set of experiments *E. coli* K12 and EHEC samples were subject to 11 and 10 runs respectively, while the wild type EHEC and *etk wzc* mutant samples in the second set of experiments were subject to three runs each. Mass spectrometry raw data was deposited in the open storage server Tranche (http://proteomecommons.org/tranche).

Raw MS spectra were processed using Proteome Discoverer (Thermo Fisher Scientific) and peak lists were searched against a NCBI protein database containing 4,094 sequences from *E. coli* K12 strain MG1655 (NC000913.2) and 5,194 sequences from EHEC O157:H7 strain EDL933 (NC002655.2 and NC007414.1) using Mascot v. 2.2 (Matrix Science) and Sequest [77]. Search criteria included trypsin as protease used separately with up to two missed cleavages allowed and carbamidomethylcysteine as static modifications. Phosphorylation of Ser/Thr/Tyr was included as variable modifications. Parent mass accuracy of 10 ppm, HCD fragmentation with mass accuracy tolerance of 0.1 Da and CID fragmentation with LTQ detection mass accuracy tolerance of 0.5 Da were used. Decoy database search was enabled to estimate levels of false discovery data. Peptides identified with an estimated false discovery rate less than 1% were used for further analysis. The location of phosphorylation sites was confirmed using PhosphoRS (Thermo Fisher Scientific).

### Bioinformatics analyses

#### Tyrosine phosphorylation site motif analysis

The Motif-X algorithm [27] was used to extract overrepresented tyrosine phosphorylation site motifs from the 512 unique pTyr sites identified in *E. coli* (Table S1). Peptide sequences with 12 amino acid residues flanking the foreground central phosphotyrosine residues were analyzed against a combined *E. coli* K12 MG1655 and EHEC O157:H7 EDL933 proteomic background with occurrences of 10 and a significance threshold level of 0.001. Parameters used for motif extraction from 7551 non-redundant phosphotyrosine sites in human proteins available in the Human Protein Reference Database [30] were: foreground central residue Y, width 13 residues, occurrences 20, significance 0.000001 and the human proteome as background. The motifs were visualized by probability logos, where the fixed tyrosine residues within the motifs are drawn at 100% height and the remaining residues according to statistical significance levels with a larger font indicating higher statistical significance. A sequence logo for the 512 pTyr sites identified from *E. coli* was generated by Phosphosite logo generator using the algorithm PSP production (Cell signaling Technology).

#### Functional classification of phosphotyrosine proteins

Phosphotyrosine proteins were manually classified into functional categories according to EcoCyc Multi-dimensional functional annotation [78].

#### Closeness centrality of phosphotyrosine proteins in the metabolic network

The protein-centric metabolic network of *E. coli* K12 and EHEC O157:H7 were extracted respectively from the EcoCyc and EcoO157Cyc flat-files as described elsewhere [79]. Briefly, if one enzyme (node) produces a compound that serves as substrate for another enzyme an edge is established between those two nodes. Highly connected compounds such as water, protons and ATP were discarded in the network reconstruction. The giant component of the reconstructed network was used to determine the closeness centrality of each node using the *igraph* library of R [80]. The non-parametric Kolmogorov-Smirnov test in R [80] was applied to determine whether the distributions of pTyr and non-pTyr proteins are significantly different in the metabolic network with *p values*≤0.05 considered statistical significant. Graph layouts were produced using *organic layout* in Cytoscape [81].

### Functional analysis

#### Western blot analysis

Levels of T3SS related proteins were determined from whole cell lysates and culture supernatants of wild type TUV93-0 and its sspA mutant derivatives containing the vector pSec10*, pAMH251 and pAMH252 expressing wild type SspA and SspAY92F respectively. Cultures were grown in DMEM at 37°C to a density of OD_600_ ∼1. Total cellular protein was precipitated with with 5% (vol/vol) trichloric acid (TCA), washed with acetone, resuspended in 1× SDS Blue loading buffer (New England Biolabs). Proteins in culture supernatants were precipitated by 10% TCA and 0.025% sodium deoxycholate along with 100 ng of GroEL (Axxora), which served as internal control for protein precipitation. Notable GroEL were not detected in precipitated culture supernatants unless it was added to the sample. Proteins were resolved on a 4–20% Tris-HCl Criterion protein gel (BioRad) and transferred onto an Immobilon-FL polyvinylidene difluoride membrane (Millipore) using a Trans-Blot SD Semi-Dry Transfer Cell (BioRad). The membrane was blocked in Odyssey blocking buffer (Li-Cor Biosciences), exposed to polyclonal antibodies specific to EspA, EspB,Tir, SspA [53] and GroEL (Sigma), and subsequently to Alexa Fluor 680-conjugated goat anti-rabbit (Invitrogen). Proteins were visualized using an Odyssey Infrared Imaging System with application software version 3.0 (Li-Cor Biosciences) as recommended. The western analyses were carried out on at least four independent samples per strain with similar results.

#### Fluorescent actin staining assay

The ability of EHEC O157:H7 TUV93-0 wild type, *sspA, etk*, *wzc* and *etk wzc* mutant derivatives thereof to form A/E lesions on HeLa cell monolayers was evaluated using the fluorescent actin staining assay (FAS) [54]. Bacterial strains were inoculated from single colonies on LB agar plates into tryptic soy broth containing antibiotics if necessary and grown statically for 18 h at 37°C. Semiconfluent HeLa cell monolayers grown on glass coverslips to ∼80% confluence were co-cultured with an initial number of ∼2×10^7^ bacteria in DMEM supplemented with 2% FBS. At 5 h post infection the monolayers were fixed in 4% formamide, washed three times with 1× PBS, permeabilized with 0.1% Triton X-100 in 1× PBS, and F-actin was stained with Alexa Fluor 488 phalloidin (Invitrogen) diluted 1∶50. Coverslips were mounted on slides using Prolong Gold antifade reagent (Invitrogen). The FAS assay was carried out independently at least three times for each strain with and results obtained were similar. Samples were visualized using an AxioSkop microscope equipped with a 40× objective and images were captured with an AxioCam MR3 digital camera using AxioVision v 4.8 software (Carl Zeiss MicroImaging Inc).

## Supporting Information

Figure S1 **Representative MS/MS spectra of phosphotyrosine peptides identified from** ***E. coli*** **.** (A) ASTApYSYGYNYYGYSpYSEKE from the tyrosine protein kinase Etk and (B) GAEpYMVDFLPK from the nitrogen regulatory protein II, GlnK. The peaks corresponding to y and b fragment ions are indicated in the MS/MS spectra along with that of the phosphotyrosine immonium ion (Imm(pY)). The position of the phosphotyrosine residue is indicated as pY.
(TIF)

Figure S2 **Phosphorylation of the tyrosine kinase Etk in EHEC O157:H7 and** ***E. coli*** **K12.** Proteins immunoprecipitated from EHEC O157:H7 and *E. coli* K12 cell lysates using the phosphotyrosine-specific antibody 4G10 were resolved by SDS-PAGE followed by coomassie staining (lanes 1–2) and western analysis using antibody 4G10 (lanes 3–4). A protein band abundant in the EHEC O157:H7 sample that was identified as Etk by MS-based identification is indicated.
(TIF)

Figure S3 **Phosphotyrosine site motifs identified among human proteins.** Motif-Χ analysis of 7551 non-redundant phosphotyrosine sites from human proteins available in the Human Protein Reference Database. Probability logos of phosphotyrosine site motifs were generated by considering 12 residues surrounding the phosphotyrosine residue (*p value*<0.000001). Site motif consensus sequences with variable residues indicated as x and the number proteins containing each motif are indicated.
(TIF)

Figure S4 **Phosphomimetic SspA mutants positively affect SspA-mediated regulation of LEE expression and T3SS.** The abundance of LEE-encoded proteins in whole cell lysates (lanes 1–6) and their abundance in culture supernatants (lanes 7–12) from cultures of wild type EHEC O157:H7 and isogenic *sspA* mutants were determined by western analyses as described in *SI Material and Methods*. Strains tested were the *sspA* mutant containing the vector control pSec10*, the *sspA* mutant expressing wild type SspA from p*sspA* and mutant SspA from pSspAY92F (SspA Y92F), pSspAY92D (SspAY92D) and pSspAY92E (SspAY92E). The phosphomimetic SspA mutants are SspAY92D and SspAY92E. EspA, EspB, SspA and GroEL were detected using polyclonal antisera against the respective proteins. GroEL served as an internal control for the total amount of protein in cell samples.
(TIF)

Figure S5 **The absence of tyrosine kinases Etk and Wzc does not alter metabolic and virulence-associated phenotypes of EHEC O157:H7.** (A) The absence of tyrosine kinases Etk and Wzc does not alter type III system (T3SS)-related phenotypes. The abundance of the T3SS-encoded translocon proteins EspA and EspB in whole cell lysates (lanes 1–4) and in culture supernatants (lanes 5–8) was determined in wild type EHEC O157:H7 (lanes 1 and 5), *wzc* (lanes 2 and 6), *etk* (lanes 3 and 7) and *etk wzc* (lanes 4 and 8) mutant backgrounds grown in DMEM at 37°C to OD_600_∼1. EspA and EspB were detected by western blot analyses using antisera specific to the respective proteins, whereas the detection of GroEL served as an internal loading control. (B) EHEC O157:H7 metabolism is unaffected by the lack of tyrosine kinases Etk and Wzc. Comparative metabolic profiling of EHEC O157:H7 WT and *etk wzc* double mutant strains grown on various carbon, nitrogen, sulfur and phosphorus sources was carried out using Biolog Phenotype Microarray PM plates 1–5 (upper panel) and 6–8 (lower panel). Growth profile overlays from WT (red trace) and the *etk wzc* mutant (green trace) with yellow indicating similar growth kinetics are shown.
(TIF)

Figure S6 **BY kinase candidates identified by structure-based** ***in silico*** **analysis.** (A) Structural superposition of Etk (3CIO, blue) and ParA (3EZ6, green) generated using the structural comparison program Dali [82]. (B) Superposition of ParA tertiary structure (green) and a ParA-based SopA model (blue) with the conserved Walker motif A, B and A′ residues indicated. (C) Structural superposition of *E. coli* Etk (green) and SopA (cyan) including Walker motif A, B and A′ residues and Etk Tyr574/Arg614 residues important for kinase activity. Residues Tyr153 and Arg346 of SopA located within a 4Å range are indicated by a broken circle.
(TIF)

Table S1 **Datasets for phosphotyrosine profiling of EHEC O157:H7 and** ***E. coli*** **K12.** Lists of unique phosphopeptides, unique phosphotyrosine peptides and the corresponding phosphotyrosine proteins for EHEC O157:H7 strain TUV93-0 (worksheets 1–3), *E. coli* K12 strain MG1655 (worksheets 4–6), and the combined dataset of the two strains (worksheets 7–8). Datasets for the second set of phosphotyrosine profiling experiments of the EHEC O157:H7 *etk wzc* double mutant (worksheets 9–11) and the EHEC O157:H7 strain that was run in parallel with this double mutant (worksheets 12–14) are listed. Peptides identified with an estimated false discovery rate less than 1% are included.
(XLS)

Table S2 **Conservation of identified phosphotyrosine sites in bacterial proteomes.** The 512 phosphotyrosine sites identified in *E. coli* were mapped to the proteomes of the following 16 bacterial strains: *Shigella flexneri* 2a str. 301, *Salmonella enterica* subsp. enterica serovar Heidelberg str. SL476, *Klebsiella pneumoniae* 342, *Yersinia pestis* Angola, *Vibrio cholerae* O395, *Haemophilus influenzae* Rd KW20, *Pseudomonas putida* KT2440, *Pseudomonas syringae* pv. tomato str. DC3000, *Bacillus anthracis* str. A0248, *Clostridium botulinum* A str. ATCC 19397, *Clostridium perfringens* ATCC 13124, *Myxococcus xanthus* DK 1622, *Cyanobacteria yellowstone* A-prime, *Streptococcus pneumoniae* 70585, *Helicobacter pylori* 26695, and *Mycoplasma genitalium* G37. Sequences of the phosphotyrosine sites conserved in the 16 proteomes and the corresponding protein information are indicated for each strain.
(XLS)

Table S3 **Phosphotyrosine site motifs identified in** ***E. coli*** **.** Phosphotyrosine site motifs that were significantly overrepresented among the 512 unique pTyr sites identified from EHEC O157:H7 and *E. coli* K12, and 103 unique pTyr sites from the EHEC O157:H7 *etk wzc* double mutant were defined using Motif-X [83]. The sequences of the peptides for which a phosphotyrosine site motif were defined and information for the corresponding proteins are shown.
(XLS)

Table S4 **Phosphotyrosine site motifs defined from sites identified in human proteins.** Phosphotyrosine site motifs from 7551 tyrosine phosphorylation sites in human proteins that showed significant overrepresentation were defined using Motif-X [83]. Peptide sequences containing one of the 13 defined phosphotyrosine site motifs and the identity of the corresponding proteins are shown. The occurrence of each motif and the number of proteins containing the motifs are indicated.
(XLS)

Table S5 **Functional classification of identified phosphotyrosine proteins.** According to EcoCyc Multi-dimensional functional annotation [78]. The functional classification of phosphotyrosine proteins identified in EHEC O157:H7, *E. coli* K12, and combined in the two strains are listed. The number of proteins in each functional class is indicated.
(XLS)

Table S6 **Functional enrichment of phosphotyrosine proteins.** Significantly enriched functional classes are listed for EHEC O157:H7 and *E. coli* K12 (corrected *p-value*≤0.05). The numbers of proteins and phosphotyrosine proteins in a given functional class are indicated along with the fold overrepresentation of phosphotyrosine proteins for each class.
(XLS)

Table S7 **Similarity searches for BY kinase catalytic domains to identify tyrosine kinase candidates.** Structure-based similarity searches of the catalytic domain of BY kinases Etk and Wzc against the EHEC O157:H7 and *E. coli* K12 proteomes carried out using PSI-BLAST [84] with an E value cut-off value of 10^−4^.
(DOC)

Table S8 **Remote structural homology detection between Etk, Wzc, SopA, MinD and ParA.** Fold recognition analyses of the fold of nucleotide-binding proteins characteristic for Etk and Wzc were carried out using HHpred [85].
(DOC)

Table S9 **Oligonucleotides used for the strain and plasmid constructions.**
(DOC)

Text S1 **Supporting materials and methods.**
(DOC)
