# Supplementary figures and images for: The Escherichia coli Phosphotyrosine Proteome Relates to Core Pathways and Virulence
Source: PLoS Pathog. 2013 Jun 13;9(6):e1003403. doi: 10.1371/journal.ppat.1003403 (PMC3681748; doi:10.1371/journal.ppat.1003403)

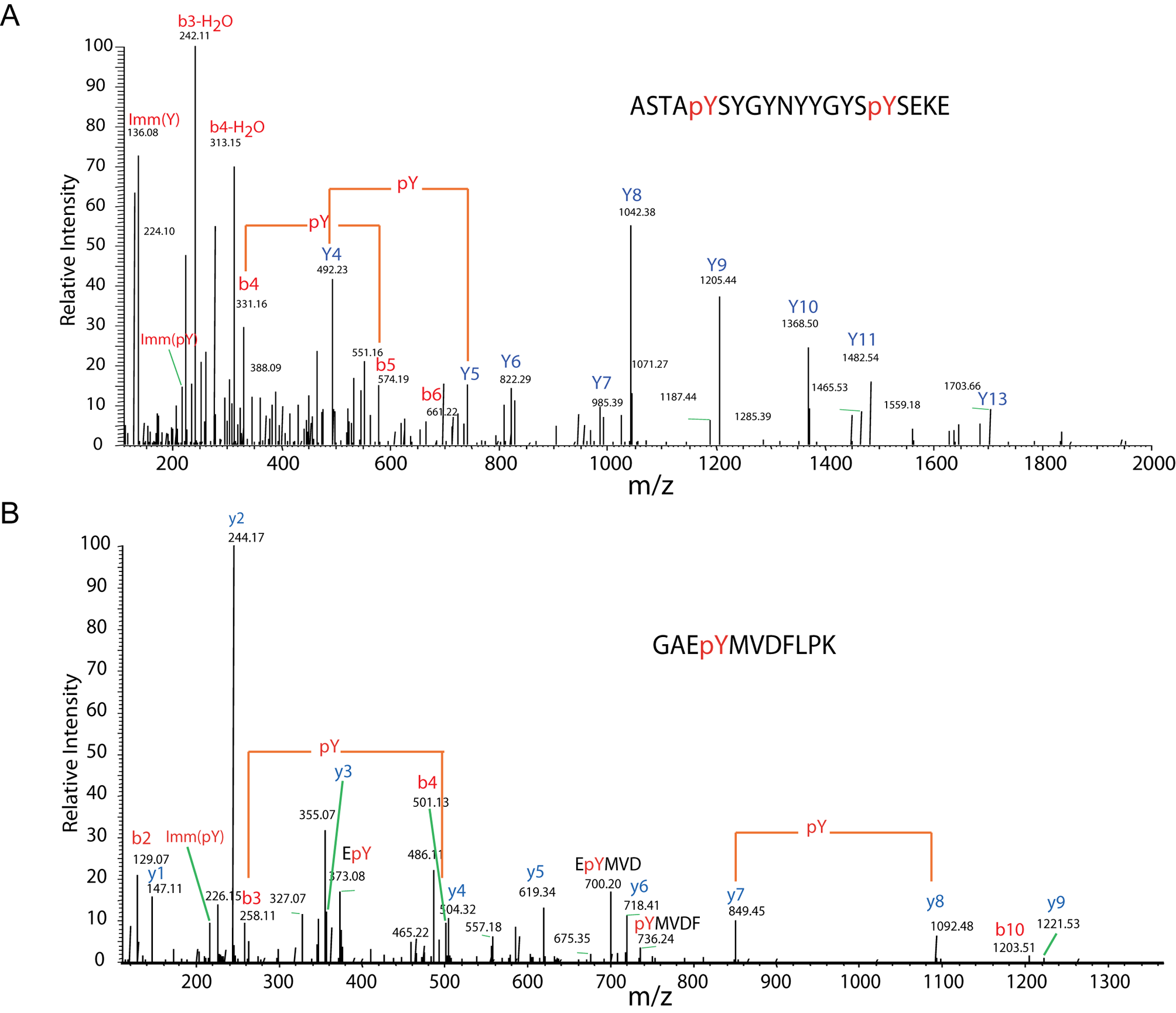

Supplement: Figure S1 — Representative MS/MS spectra of phosphotyrosine peptides identified from E. coli . (A) ASTApYSYGYNYYGYSpYSEKE from the tyrosine protein kinase Etk and (B) GAEpYMVDFLPK from the nitrogen regulatory protein II, GlnK. The peaks corresponding to y and b fragment ions are indicated in the MS/MS spectra along with that of the phosphotyrosine immonium ion (Imm(pY)). The position of the phosphotyrosine residue is indicated as pY. (TIF) [file ppat.1003403.s001.tif]

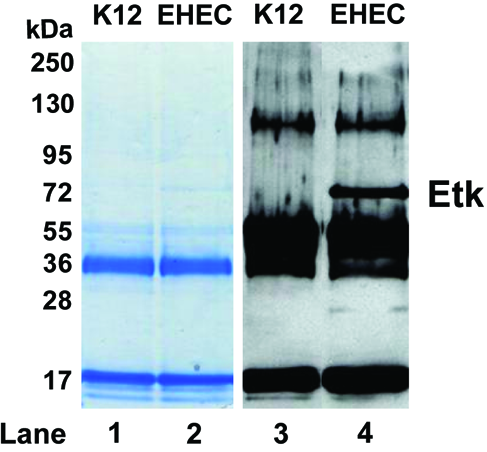

Supplement: Figure S2 — Phosphorylation of the tyrosine kinase Etk in EHEC O157:H7 and E. coli K12. Proteins immunoprecipitated from EHEC O157:H7 and E. coli K12 cell lysates using the phosphotyrosine-specific antibody 4G10 were resolved by SDS-PAGE followed by coomassie staining (lanes 1–2) and western analysis using antibody 4G10 (lanes 3–4). A protein band abundant in the EHEC O157:H7 sample that was identified as Etk by MS-based identification is indicated. (TIF) [file ppat.1003403.s002.tif]

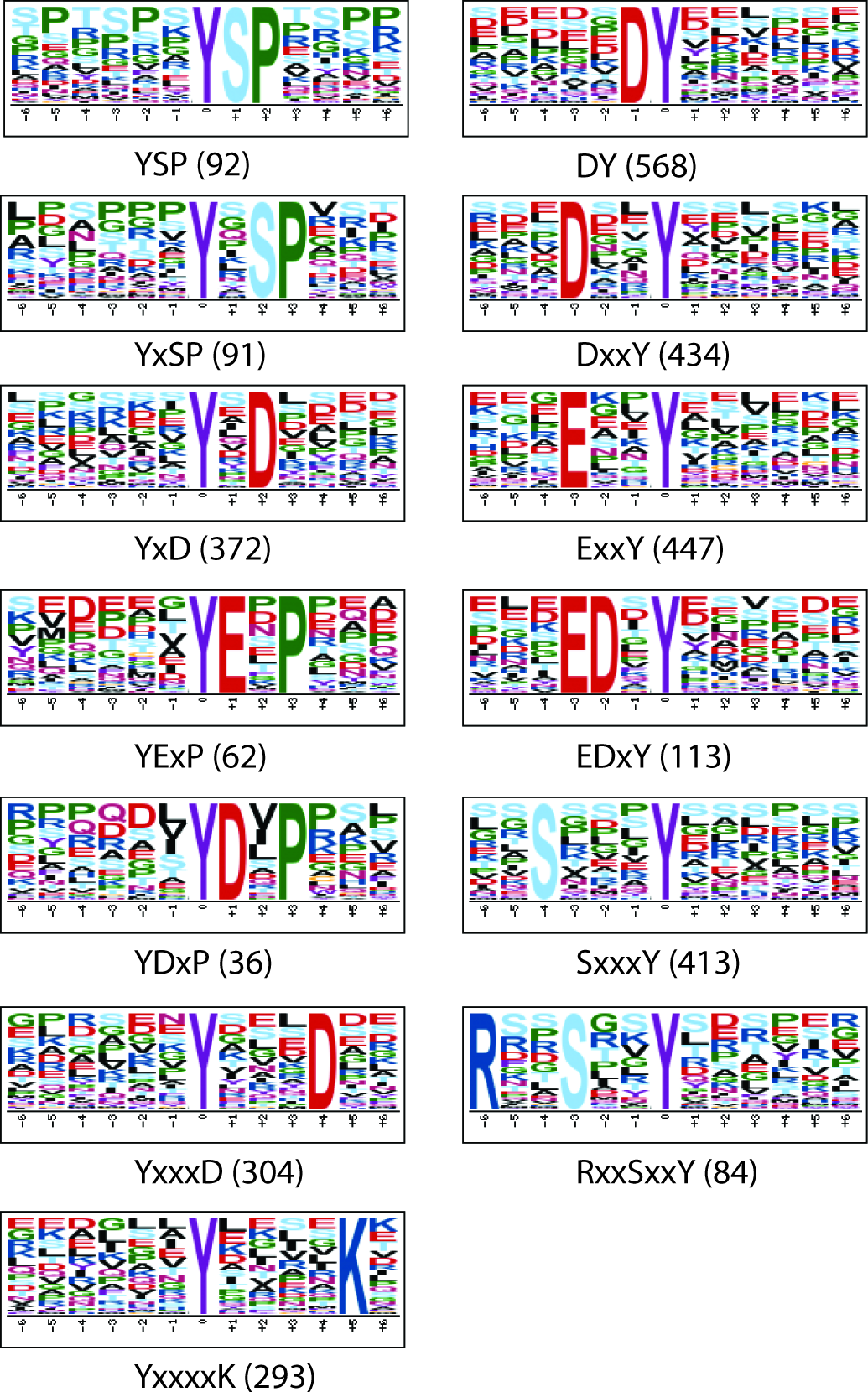

Supplement: Figure S3 — Phosphotyrosine site motifs identified among human proteins. Motif-Χ analysis of 7551 non-redundant phosphotyrosine sites from human proteins available in the Human Protein Reference Database. Probability logos of phosphotyrosine site motifs were generated by considering 12 residues surrounding the phosphotyrosine residue (p value<0.000001). Site motif consensus sequences with variable residues indicated as x and the number proteins containing each motif are indicated. (TIF) [file ppat.1003403.s003.tif]

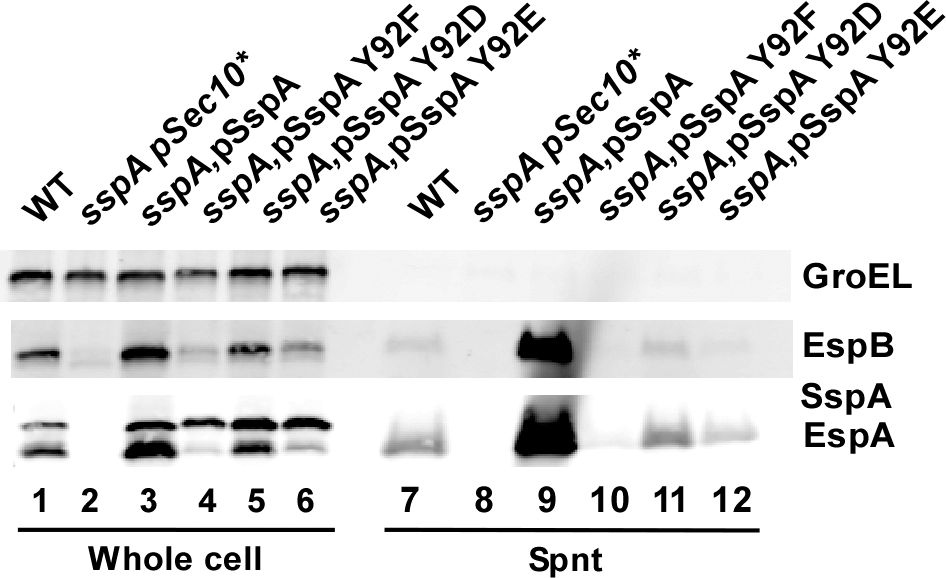

Supplement: Figure S4 — Phosphomimetic SspA mutants positively affect SspA-mediated regulation of LEE expression and T3SS. The abundance of LEE-encoded proteins in whole cell lysates (lanes 1–6) and their abundance in culture supernatants (lanes 7–12) from cultures of wild type EHEC O157:H7 and isogenic sspA mutants were determined by western analyses as described in SI Material and Methods. Strains tested were the sspA mutant containing the vector control pSec10*, the sspA mutant expressing wild type SspA from psspA and mutant SspA from pSspAY92F (SspA Y92F), pSspAY92D (SspAY92D) and pSspAY92E (SspAY92E). The phosphomimetic SspA mutants are SspAY92D and SspAY92E. EspA, EspB, SspA and GroEL were detected using polyclonal antisera against the respective proteins. GroEL served as an internal control for the total amount of protein in cell samples. (TIF) [file ppat.1003403.s004.tif]

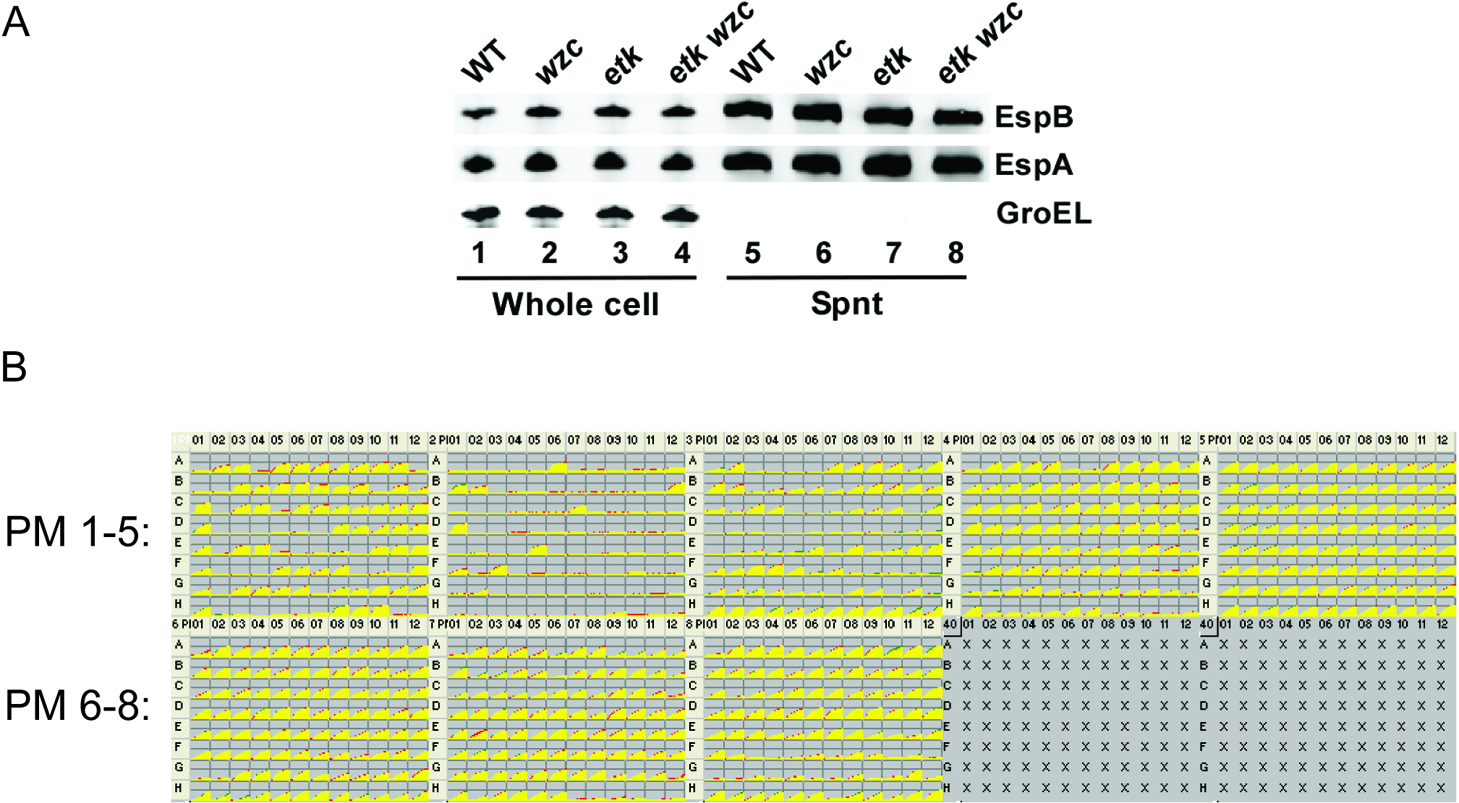

Supplement: Figure S5 — The absence of tyrosine kinases Etk and Wzc does not alter metabolic and virulence-associated phenotypes of EHEC O157:H7. (A) The absence of tyrosine kinases Etk and Wzc does not alter type III system (T3SS)-related phenotypes. The abundance of the T3SS-encoded translocon proteins EspA and EspB in whole cell lysates (lanes 1–4) and in culture supernatants (lanes 5–8) was determined in wild type EHEC O157:H7 (lanes 1 and 5), wzc (lanes 2 and 6), etk (lanes 3 and 7) and etk wzc (lanes 4 and 8) mutant backgrounds grown in DMEM at 37°C to OD600∼1. EspA and EspB were detected by western blot analyses using antisera specific to the respective proteins, whereas the detection of GroEL served as an internal loading control. (B) EHEC O157:H7 metabolism is unaffected by the lack of tyrosine kinases Etk and Wzc. Comparative metabolic profiling of EHEC O157:H7 WT and etk wzc double mutant strains grown on various carbon, nitrogen, sulfur and phosphorus sources was carried out using Biolog Phenotype Microarray PM plates 1–5 (upper panel) and 6–8 (lower panel). Growth profile overlays from WT (red trace) and the etk wzc mutant (green trace) with yellow indicating similar growth kinetics are shown. (TIF) [file ppat.1003403.s005.tif]

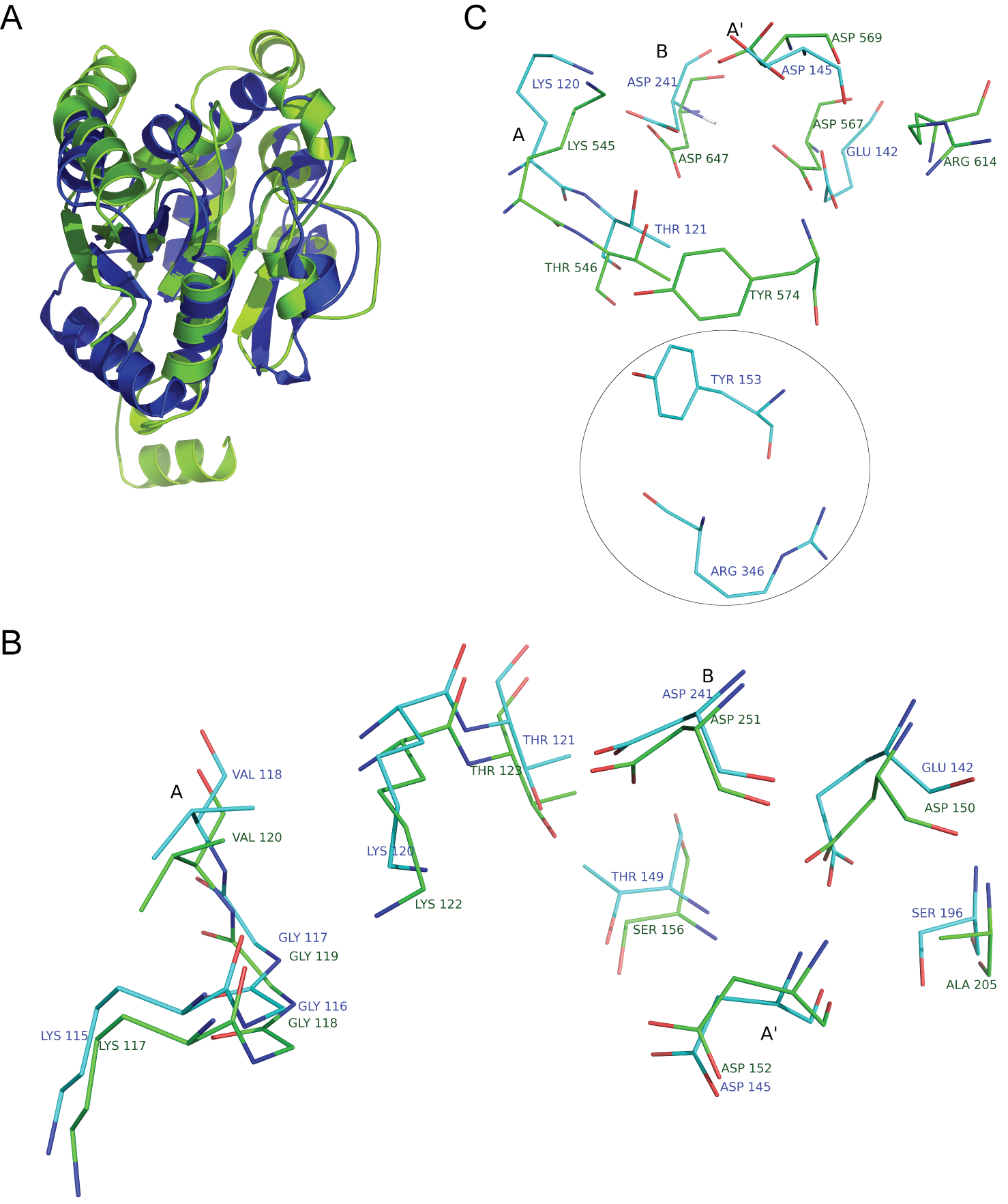

Supplement: Figure S6 — BY kinase candidates identified by structure-based in silico analysis. (A) Structural superposition of Etk (3CIO, blue) and ParA (3EZ6, green) generated using the structural comparison program Dali [82]. (B) Superposition of ParA tertiary structure (green) and a ParA-based SopA model (blue) with the conserved Walker motif A, B and A′ residues indicated. (C) Structural superposition of E. coli Etk (green) and SopA (cyan) including Walker motif A, B and A′ residues and Etk Tyr574/Arg614 residues important for kinase activity. Residues Tyr153 and Arg346 of SopA located within a 4Å range are indicated by a broken circle. (TIF) [file ppat.1003403.s006.tif]
