# Supplementary material for: The Escherichia coli Phosphotyrosine Proteome Relates to Core Pathways and Virulence
Source: PLoS Pathog. 2013 Jun 13;9(6):e1003403. doi: 10.1371/journal.ppat.1003403 (PMC3681748; doi:10.1371/journal.ppat.1003403)
Supplement: Table S7 — Similarity searches for BY kinase catalytic domains to identify tyrosine kinase candidates. Structure-based similarity searches of the catalytic domain of BY kinases Etk and Wzc against the EHEC O157:H7 and E. coli K12 proteomes carried out using PSI-BLAST [84] with an E value cut-off value of 10−4. (DOC) [file ppat.1003403.s013.doc]

**TABLE S7**

Similarity searches for BY kinase catalytic domains to identify tyrosine kinase candidates

| Query | Hit  Accession | Hit*  Protein | % Identity | Query start | Query end | Hit  start | Hit  end | E-value | % Query coverage |
| --- | --- | --- | --- | --- | --- | --- | --- | --- | --- |
| Etk | NP_309164.1 | Etk (EHEC) | 100 | 1 | 277 | 450 | 726 | 2e-166 | 100 |
| Etk | YP_001729958 | Etk (K12) | 100 | 1 | 277 | 450 | 726 | 2e-166 | 100 |
| Etk | YP_001731010.1 | Wzc (K12) | 53 | 2 | 276 | 451 | 720 | 5e-86 | 99 |
| Etk | NP_310892.2 | Wzc (EHEC) | 53 | 2 | 276 | 451 | 720 | 5e-86 | 99 |
| Etk | NP_052640.1 | SopA (EHEC) | 22 | 63 | 211 | 84 | 254 | 3e-3 | 53 |
| Etk | P62556 | SopA (K12) | 22 | 63 | 211 | 84 | 254 | 3e-3 | 53 |
| Wzc | NP_310892.2 | Wzc (EHEC) | 100 | 1 | 271 | 450 | 720 | 3e-162 | 100 |
| Wzc | YP_001731010.1 | Wzc (K12) | 100 | 1 | 271 | 450 | 720 | 3e-162 | 100 |
| Wzc | YP_001729958.1 | Etk (K12) | 54 | 2 | 254 | 451 | 708 | 6e-83 | 93 |
| Wzc | NP_309164.1 | Etk (EHEC) | 54 | 2 | 254 | 451 | 708 | 6e-83 | 93 |
| Wzc | NP_052640.1 | SopA (EHEC) | 25 | 58 | 200 | 84 | 248 | 3e-4 | 52 |
| Wzc | P62556 | SopA (K12) | 25 | 58 | 200 | 84 | 248 | 3e-4 | 52 |

*EHEC and K12 refer to *E. coli* O157:H7 strain EDL933 and *E. coli* K12 strain MG1655 respectively
