# Supplementary material for: The Escherichia coli Phosphotyrosine Proteome Relates to Core Pathways and Virulence
Source: PLoS Pathog. 2013 Jun 13;9(6):e1003403. doi: 10.1371/journal.ppat.1003403 (PMC3681748; doi:10.1371/journal.ppat.1003403)
Supplement: Table S8 — Remote structural homology detection between Etk, Wzc, SopA, MinD and ParA. Fold recognition analyses of the fold of nucleotide-binding proteins characteristic for Etk and Wzc were carried out using HHpred [85]. (DOC) [file ppat.1003403.s014.doc]

**TABLE S8**

Remote structural homology detection between Etk, Wzc, SopA, MinD and ParA

| Query HMM | Hit HMM | E-value |
| --- | --- | --- |
| Etk kinase | MinD, PDB id: 1G3Q | 4.9e28 |
| Etk Kinase | ParA, PDB id: 3EZ2 | 4.3e23 |
| Wzc kinase | MinD, PDB id: 1G3Q | 9.2e29 |
| Wzc kinase | ParA, PDB id: 3EZ2 | 2.7e23 |
| SopA | Etk, PDB id: 3CIO | 6.7e24 |
| SopA | MinD, PDB id: 1G3Q | 9.7e31 |
| SopA | ParA, PDB id: 3EZ2 | 0 |
