# Supplementary material for: The Escherichia coli Phosphotyrosine Proteome Relates to Core Pathways and Virulence
Source: PLoS Pathog. 2013 Jun 13;9(6):e1003403. doi: 10.1371/journal.ppat.1003403 (PMC3681748; doi:10.1371/journal.ppat.1003403)
Supplement: Table S9 — Oligonucleotides used for the strain and plasmid constructions. (DOC) [file ppat.1003403.s015.doc]

**TABLE S9**

Oligonucleotides used for the strain and plasmid construction

| **Name** | **Oligo sequence (5’ to 3’)** |
| --- | --- |
|  |  |
| AH1166 | CCCAAATGTCGGGTATTGCTCAGGAGGTTTCTTTCATGATGTCCGGGGATCCGTCGACCT |
| AH1167 | GATTAACTCCGGCCCAGACGCATTTCACGTTCTGCTTCAGTGTAGGCTGGAGCTGCTTCG |
| AH1168 | CACCGTCGACGTAAAGCACGTCGTCGTCCGCA |
| AH1169 | CACCGAAGCTTCAACCACTCATAGAATGCACGCAGCA |
| AH1171 | CTGATGCCTGTTTTCCCGGTAGCTCGCGGT |
| AH1172 | ACCGCGAGCTACCGGGAAAACAGGCATCAG |
| AH1206 | CTGATGCCTGTTGACCCGGTAGCTCGCGGT |
| AH1207 | ACCGCGAGCTACCGGGTCAACAGGCATCAG |
| AH1208 | CTGATGCCTGTTGAACCGGTAGCTCGCGGT |
| AH1209 | ACCGCGAGCTACCGGTTCAACAGGCATCAG |
| K6486 | AATGGGCGAAGCGCCTCAGCCGGTAATTTGAGTTTATAAAATACGATG TCCGGGGATCCGTCGACCT |
| K6487 | CCGGAAAAGGCGTTCACGCCGCATCCGGTCACTTTACCTTCAGTTTTAGTGTAGGCTGGAGCTGCTTCG |
| K6488 | CGATCTGATTCTGGCAATGGAGTC |
| K6489 | GCAGGATGTGAAGAGCGAAATG |
| K6490 | GAAATCGATATTGGTCGCCTGGTCGGCACCGTCATTGAAGCGCGCATG TCCGGGGATCCGTCGACCT |
| K6491 | TGAGCGCCTTATCCGGCCTACGGGGCGGTGCGAATGCAGGCCTCGTTAGTGTAGGCTGGAGCTGCTTCG |
| K6492 | CATATCGAACGCTTATGCGAGATG |
| K6493 | CGATGATCATCTCCCAGTTGCTG |
