# Supplementary material for: The Escherichia coli Phosphotyrosine Proteome Relates to Core Pathways and Virulence
Source: PLoS Pathog. 2013 Jun 13;9(6):e1003403. doi: 10.1371/journal.ppat.1003403 (PMC3681748; doi:10.1371/journal.ppat.1003403)
Supplement: Text S1 — Supporting materials and methods. (DOC) [file ppat.1003403.s016.doc]

Supporting information

SUPPLEMENTARY MATERIALS AND METHODS:

**Evolutionary conservation of phosphotyrosine sites**

Protein sequences were obtained of the following 16 genomes: *Bacillus anthracis* A0248 (NC012659), C*lostridium botulinum* A ATCC 19397 (NC009697.1), *Clostridium perfringens* ATCC13124 (NC008261.1), *Cyanobacteria yellowstone* A-prime (NC007775.1), *Haemophilus influenzae* KW20 Rd (NC000907.1), *Helicobacter pylori* 26695 (NC000915.1), *Klebsiella pneumoniae* 342 (NC011283.1), *Mycoplasma genitalium* G37 (NC000908.2), *Myxococcus xanthus* DK 1622 (NC008095.1), *Pseudomonas putida* KT2440 (NC002947.3), *Pseudomonas syringae* DC3000 (NC004578.1), *Salmonella enterica* subsp. enterica serovar Heidelberg str. SL476 (NC011083.1), *Streptococcus pneumoniae* 70585 (NC012468.1), *Vibrio cholerae* O395 (NC009456.1 and NC009457.1) and *Yersinia pestis* Angola (NC010159.1) from JCVI and the *Shigella flexneri* 2a str. 301 (NC004337.2) genome sequences from NCBI genome using the GenBank FTP site. We considered 5 amino acid residues upstream and downstream of the 512 unique phosphotyrosine sites identified combined from EHEC O157:H7 and *E. coli* K12 (Table S1), and mapped these sequences to protein sequences of the 16 bacterial genomes listed above using in-house Python scripts. Only exact matches between the 11 amino residue-long peptides and proteins from the 16 bacterial strains were considered. Positions of phosphotyrosine sites were parsed from the corresponding protein sequences and total count of proteins from each genome were taken using in-house Python scripts. We parsed motifs that were present in at least 14 out of 16 genomes and considered these as highly conserved.

**Functional enrichment of phosphotyrosine proteins**

Functional enrichment of phosphotyrosine proteins identified from *E. coli* K12 and EHEC O157:H7 was determined based on three databases of functional annotation classes: Gene Ontology (GO) biological processes [1], EcoCyc/Transporter Classification Database (TCDB) for membrane transport proteins [2], and EcoCyc metabolic pathways for *E. coli* K12 [3] and BioCyc metabolic pathways for EHEC O157:H7 [4,5]. GO annotations were assigned to 134 of 290 and 100 of 119 phosphotyrosine proteins respectively from EHEC O157:H7 and *E. coli* K12 when considering GO terms with at least five and less than 150 members. Functional classes represented in each set of phosphotyrosine proteins were contrasted against their corresponding full proteome annotations. We used Fisher's exact test to obtain *p values* that reflect the likelihood of obtaining exactly the observed number of phosphotyrosine proteins belonging to each functional class in which they are annotated, given the total number proteins in such class in the full proteome, and the total number of phosphotyrosine proteins identified. The resulting *p values* were corrected for multiple-hypothesis testing using the Benjamini and Hochberg method [6]. Functional classes with a corrected *p value* ≤ 0.05 were considered significantly enriched. The fold ratio of overrepresentation of hits for each class (*i*) was computed using the formula ‘Ratio(*i*) = (a/n)/(m/N)', where 'a' is the number of hits in (*i*), 'n' is the total number of hits, 'm' is the total number of proteins in (*i*), and 'N' is the total number of proteins in the proteome.

**Construction of phosphomimetic mutants of SspA Tyr92**

Plasmids expressing phosphomimetics SspA mutants p*sspA*Y92D (pAMH265) and p*sspA*Y92E (pAMH266) were constructed by site directed mutagenesis of *sspA* encoded by pAMH251 (this study) using primer sets AH1206/AH1207 and AH1208/AH1209, respectively, and the QuickChange XL Site-Directed Mutagenesis Kit (Agilent) as recommended by the manufacturer. Oligo sequences used for plasmid constructions are listed in table S9.

## Western blot analysis

Levels of type three secretion system related proteins were determined from whole cell lysates and culture supernatants of EHEC O157:H7 strain TUV93-0 and mutant derivatives thereof. Overnight cultures were diluted 1:1000 in DMEM and grown aerobically at 37ºC to an optical density value at 600 nm (OD_600_) ~1. Total protein was precipitated from respectively whole cell and culture supernatants fractions with 5% (vol/vol) trichloric acid, washed with 80% acetone, resuspended in 1X SDS Blue loading buffer (New England Biolabs) and protein samples equivalent to 0.03 OD_600_ units of culture were resolved on a 10% SDS-PAGE. Protein was transferred onto an Immobilon-FL polyvinylidene difluoride membrane (Millipore) using a Trans-Blot SD Semi-Dry Transfer Cell (BioRad). The membrane was blocked in Odyssey blocking buffer (Li-Cor Biosciences), exposed to polyclonal antibodies specific to EspA, EspB, SspA and GroEL (Sigma), and subsequently to Alexa Fluor 680-conjugated goat anti-rabbit (Invitrogen). Proteins were visualized using an Odyssey Infrared Imaging System with application software version 2.1 (Li-Cor Biosciences) as recommended. Levels of Tyr phosphorylated proteins in *E. coli* K12 and EHEC were analyzed by pTyr immunoprecipitation using agarose-bound anti-phosphotyrosine 4G10 antibodies followed by Western blot analysis using a HRP-conjugated 4G10 antibody (Upstate Biotechnology).

**Biolog Phenotype Microarray analysis**

The phenotypic metabolic analyses were performed by the Biolog Phenotype Microarray (PM) Services (Hayward, CA). The metabolic profiles of EHEC wild type and the *etk wzc* double mutant were compared in duplicates of eight 96-well plates comprising eight metabolic panels (PM1 to PM8, ~800 test conditions). The PM analysis was carried out using an OmniLog instrument and standard PM testing protocols as described at [http://www.biolog.com](http://www.biolog.com/). Briefly, metabolic activity observed as bacterial growth in Biolog defined minimal medium or nitrogen, phosphorous and sulphur-free derivatives hereof for 24 h was quantified based on the reduction kinetics of the tetrazolium indicator dye [7,8]. The colorimetric redox changes from wild type and *etk wzc* mutant strains were recorded as red and green trace signals, respectively, and compared using OmniLog v 1.5 software.

***in silico* search for BY kinase candidates**

Homology searches of the catalytic domains of Etk (residues 450-726) and Wzc (residues 450-720) against a database of EHEC O157:H7 (NC002655.2 and NC007414.1) and *E. coli* K12 (NC000913.2) proteomes were carried out using PSI-BLAST [9]. An inclusion threshold value of 10^-4^ was used to define hits in a given round of iterations to be included in a position-specific scoring matrix that was used as query in the next iteration. The resulting homologues of the query were defined using an E value cut-off of 10^-4^. Remote structural homology between Etk, Wzc, MinD, ParA and SopA were established by hidden Markov model (HMM)-HMM alignments using HHpred [10]. The HMM profile was generated by aligning full length protein sequences of MinD, ParA, SopA followed by search against a HMM profile data base generated using full length protein sequences from PDB ([www.pdb.org](http://www.pdb.org/), [11]) or protein domains as defined in the SCOP database [12].

A tertiary structure model of SopA (39-383) was generated based on the ParA structure (3EZ6) using Modeller v. 9.0 [13]. An alignment of SopA and ParA protein sequences generated by considering structural features of ParA was analyzed by Modeller using default parameters. Recognition of the three-dimensional fold of SopA was pursued using Phyre v. 2 [14] at default parameters and the fold of BY kinases Etk and Wzc as queries. Structural superpositions were generated using the structural comparison program Dali [15].

**References**

1. Barrell D, Dimmer E, Huntley RP, Binns D, O'Donovan C, Apweiler R (2009) The GOA database in 2009--an integrated Gene Ontology Annotation resource. Nucleic Acids Res 37: D396-D403.

2. Saier MH, Jr., Tran CV, Barabote RD (2006) TCDB: the Transporter Classification Database for membrane transport protein analyses and information. Nucleic Acids Res 34: D181-D186.

3. Keseler IM, Collado-Vides J, Santos-Zavaleta A, Peralta-Gil M, Gama-Castro S, Muniz-Rascado L, Bonavides-Martinez C, Paley S, Krummenacker M, Altman T, Kaipa P, Spaulding A, Pacheco J, Latendresse M, Fulcher C, Sarker M, Shearer AG, Mackie A, Paulsen I, Gunsalus RP, Karp PD (2011) EcoCyc: a comprehensive database of *Escherichia coli* biology. Nucleic Acids Res 39: D583-D590.

4. Karp PD, Ouzounis CA, Moore-Kochlacs C, Goldovsky L, Kaipa P, Ahren D, Tsoka S, Darzentas N, Kunin V, Lopez-Bigas N (2005) Expansion of the BioCyc collection of pathway/genome databases to 160 genomes. Nucleic Acids Res 33: 6083-6089.

5. Perna NT, Plunkett G, III, Burland V, Mau B, Glasner JD, Rose DJ, Mayhew GF, Evans PS, Gregor J, Kirkpatrick HA, Posfai G, Hackett J, Klink S, Boutin A, Shao Y, Miller L, Grotbeck EJ, Davis NW, Lim A, Dimalanta ET, Potamousis KD, Apodaca J, Anantharaman TS, Lin J, Yen G, Schwartz DC, Welch RA, Blattner FR (2001) Genome sequence of enterohaemorrhagic *Escherichia coli* O157:H7. Nature 409: 529-533.

6. Benjamini Y, Hochberg Y (1995) Controlling the False Discovery Rate: a Practical and Powerful Approach to Multiple Testing. J R Statist Soc B: 289-300.

7. Bochner BR, Gadzinski P, Panomitros E (2001) Phenotype microarrays for high-throughput phenotypic testing and assay of gene function. Genome Res 11: 1246-1255.

8. Zhou L, Lei XH, Bochner BR, Wanner BL (2003) Phenotype microarray analysis of *Escherichia coli* K-12 mutants with deletions of all two-component systems. J Bacteriol 185: 4956-4972.

9. Altschul SF, Madden TL, Schaffer AA, Zhang J, Zhang Z, Miller W, Lipman DJ (1997) Gapped BLAST and PSI-BLAST: a new generation of protein database search programs. Nucleic Acids Res 25: 3389-3402.

10. Soding J, Biegert A, Lupas AN (2005) The HHpred interactive server for protein homology detection and structure prediction. Nucleic Acids Res 33: W244-W248.

11. Berman HM, Westbrook J, Feng Z, Gilliland G, Bhat TN, Weissig H, Shindyalov IN, Bourne PE (2000) The Protein Data Bank. Nucleic Acids Res 28: 235-242.

12. Murzin AG, Brenner SE, Hubbard T, Chothia C (1995) SCOP: a structural classification of proteins database for the investigation of sequences and structures. J Mol Biol 247: 536-540.

13. Marti-Renom MA, Stuart AC, Fiser A, Sanchez R, Melo F, Sali A (2000) Comparative protein structure modeling of genes and genomes. Annu Rev Biophys Biomol Struct 29: 291-325.

14. Kelley LA, Sternberg MJ (2009) Protein structure prediction on the Web: a case study using the Phyre server. Nat Protoc 4: 363-371.

15. Holm L, Rosenstrom P (2010) Dali server: conservation mapping in 3D. Nucleic Acids Res 38: W545-W549.
